# Covalent Organic Framework Nanobowls as Activatable Nanosensitizers for Tumor‐Specific and Ferroptosis‐Augmented Sonodynamic Therapy

**DOI:** 10.1002/advs.202206009

**Published:** 2023-01-03

**Authors:** Shanshan Zhang, Shujun Xia, Liang Chen, Yu Chen, Jianqiao Zhou

**Affiliations:** ^1^ Department of Ultrasound Ruijin Hospital Shanghai Jiaotong University School of Medicine Shanghai 200025 P. R. China; ^2^ Materdicine Lab School of Life Sciences Shanghai University Shanghai 200444 P. R. China

**Keywords:** activatable therapy, biomedicine, covalent organic frameworks, ferroptosis, sonodynamic therapy

## Abstract

Covalent organic frameworks (COFs) have attracted increasing attention for biomedical applications. COFs‐based nanosensitizers with uniform nanoscale morphology and tumor‐specific curative effects are in high demand; however, their synthesis is yet challenging. In this study, distinct COF nanobowls are synthesized in a controlled manner and engineered as activatable nanosensitizers with tumor‐specific sonodynamic activity. High crystallinity ensures an ordered porous structure of COF nanobowls for the efficient loading of the small‐molecule sonosensitizer rose bengal (RB). To circumvent non‐specific damage to normal tissues, the sonosensitization effect is specifically inhibited by the in situ growth of manganese oxide (MnO*_x_*) on RB‐loaded COFs. Upon reaction with tumor‐overexpressed glutathione (GSH), the “gatekeeper” MnO*_x_* is rapidly decomposed to recover the reactive oxygen species (ROS) generation capability of the COF nanosensitizers under ultrasound irradiation. Increased intracellular ROS stress and GSH consumption concomitantly induce ferroptosis to improve sonodynamic efficacy. Additionally, the unconventional bowl‐shaped morphology renders the nanosensitizers with enhanced tumor accumulation and retention. The combination of tumor‐specific sonodynamic therapy and ferroptosis achieves high efficacy in killing cancer cells and inhibiting tumor growth. This study paves the way for the development of COF nanosensitizers with unconventional morphologies for biomedicine, offering a paradigm to realize activatable and ferroptosis‐augmented sonodynamic tumor therapy.

## Graphical abstract

Covalent organic framework (COF) nanobowls are synthesized and rationally engineered as activatable nanosensitizers by loading small‐molecule sonosensitizers and enveloping the surface with manganese oxide. Taking advantage of glutathione‐activated sonodynamic effects and enhanced tumor accumulation, COF nanosensitizers can realize highly efficient tumor‐specific and ferroptosis‐augmented sonodynamic therapy.

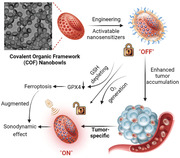

## 1 Introduction

Covalent organic frameworks (COFs) have emerged as a new class of crystalline porous materials with flexible building blocks, regular porosity, and high chemical stability.^[^ ^1^ ^]^ Extensive research has focused on the monomer design and synthetic strategies of COFs, as well as their applications in diverse fields, such as catalysis,^[^ ^2^ ^]^ gas absorption or separation,^[^ ^3^ ^]^ sensors,^[^ ^4^ ^]^ and energy storage.^[^ ^5^ ^]^ However, the application of COFs in the biomedical field is still in its infancy. One reason is that it is paramount, but challenging, to synthesize nanoscale COFs with controlled morphology to meet the requirements of systemic administration.^[^ ^6^ ^]^ Bulk COFs synthesized by solvothermal methods are generally interparticle cross‐linked agglomerations that are difficult to disperse in biological media. Mechanical exfoliation has been adopted to prepare COF nanoparticles from bulk COFs via the top‐down route; however, the time‐consuming process and uncontrolled morphology of the products still impede the broad application of COFs.^[^ ^7^ ^]^ Tuning the nanoscale morphology of COFs is anticipated to boost their physicochemical properties and performance.^[^ ^8^ ^]^ In particular, an in‐depth investigation of the structure–property–performance relationship necessitates the development of uniform COFs with well‐defined nanoarchitectures.^[^ ^9^ ^]^ To date, COFs with traditional shapes, including nanospheres and nanosheets, have been fabricated and utilized in drug delivery, phototherapy, and biosensing.^[^ ^10^ ^]^ Nevertheless, the biological effects and therapeutic applications of COFs with unconventional morphologies have rarely been explored.^[^ ^11^ ^]^ Therefore, the controlled synthesis of nanoscale COFs with distinctive morphologies and high crystallinity is of utmost significance for extending their application to specific nanotherapy.

Sonodynamic therapy (SDT) is a promising strategy for cancer treatment owing to its noninvasiveness, cost‐effectiveness, and high tissue penetration depth.^[^ ^12^ ^]^ Diverse sonosensitizers have been used to generate reactive oxygen species (ROS) to induce cell apoptosis or necrosis under ultrasound (US) stimulation.^[^ ^13^ ^]^ Nanosensitizers, primarily small molecule sonosensitizer‐encapsulated nanoformulations and inorganic nanosensitizers, can achieve favorable pharmacokinetics and therapeutic performance.^[^ ^14^ ^]^ However, the majority of currently available nanosensitizers feature “always on” sonodynamic activities, leading to a high risk of off‐target toxicity and undesired damage to normal tissues.^[^ ^15^ ^]^ Typical strategies used in activatable photodynamic therapy might not be applicable for SDT, and very few studies have attempted to overcome this drawback.^[^ ^16^ ^]^ Furthermore, another challenge encountered by nanosensitizers is that the killing effects of sonodynamic‐originated ROS are compromised by intracellular glutathione (GSH).^[^ ^17^ ^]^ In this regard, an additional killing mechanism should be introduced to synergize with SDT to improve therapeutic efficacy.^[^ ^18^ ^]^ Taken together, we speculate that functionalized COFs with distinctive morphologies can be engineered as activatable nanosensitizers to combine tumor‐specific SDT and synergistic therapeutic functions, enabling highly efficient nanodynamic therapy with specific selectivity and minimal side effects.^[^ ^19^ ^]^

**Scheme 1:**
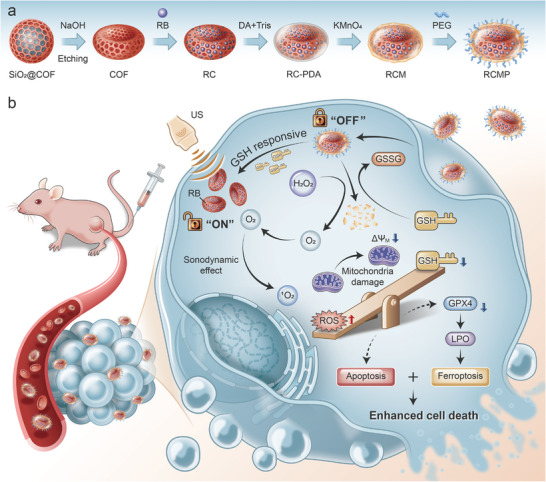
Schematic illustration for the preparation and therapeutic application of the activatable nanosensitizers RCMP. a) The synthetic process of RCMP. b) Schematic illustration of RCMP for activatable sonodynamic tumor therapy and ferroptosis. The bowl‐shaped nanosensitizers can realize enhanced tumor accumulation and cancer cell uptake. Subsequently, the overexpressed intracellular GSH serves as a key to unlock the sonodynamic activity of the nanosensitizers, accompanied by oxygen evolution and GSH depletion. The integration of augmented SDT and synergistic ferroptosis effectively enhances cancer cell death. RC: RB@COF; RCM: RB@COF‐MnO*_x_*; RCMP: RB@COF‐MnO*_x_*‐PEG; GSH: glutathione; COF: covalent organic frameworks; RB: rose bengal; PEG: polyethylene glycol.

In this paper, we report the controlled synthesis and rational functionalization of COF nanobowls to realize activatable and ferroptosis‐boosted SDT (**Scheme** **1**). The COF nanobowls were synthesized using a hard‐template method under mild conditions and showed high crystallinity and uniform morphologies. The porous characteristics of COFs made them desirable vehicles for loading the small‐molecule sonosensitizer rose bengal (RB). Subsequently, MnO*_x_* was employed as a tumor‐specific “gatekeeper” to seal the RB‐loaded COF nanobowls via a polydopamine‐mediated redox reaction, without affecting the highly crystalline nature of COFs. After modification with polyethylene glycol (PEG), the obtained nanosensitizers (RB@COFs‐MnO*_x_*‐PEG, designated as RCMP) exhibited significantly suppressed sonodynamic activity under normal physiological conditions. Contrarily, high‐concentration GSH in tumor cells caused the collapse of surface MnO*_x_*, resulting in the transformation of RCMP from “off” to “on” states to implement the sonodynamic process in cancer cells. The catalytic effects of MnO*_x_* also facilitated intracellular oxygen evolution and GSH depletion, improving sonodynamic therapeutic efficacy. The boosted intracellular ROS, combined with GSH consumption, efficiently induced ferroptosis in cancer cells via GSH peroxidase 4 (GPX4) inactivation and lipid peroxidation (LPO) accumulation. In addition, bowl‐shaped COF nanosensitizers outperformed their spherical counterparts in terms of cellular uptake and tumor accumulation. Consequently, the synergistic sonodynamic and ferroptosis effects and morphology‐enhanced performance of RCMP amplified its efficacy in killing cancer cells and preventing osteosarcoma progression. This study not only presents activatable COF nanosensitizers that can achieve high synergistic efficacy of tumor‐specific SDT and ferroptosis but also offers insight into the controlled fabrication and biological effects of nanoscale COFs with unconventional morphology and high crystallinity.

## 2 Results and Discussion

### 2.1 Synthesis and Characterization of Functionalized COF Nanobowls

**Figure 1:**
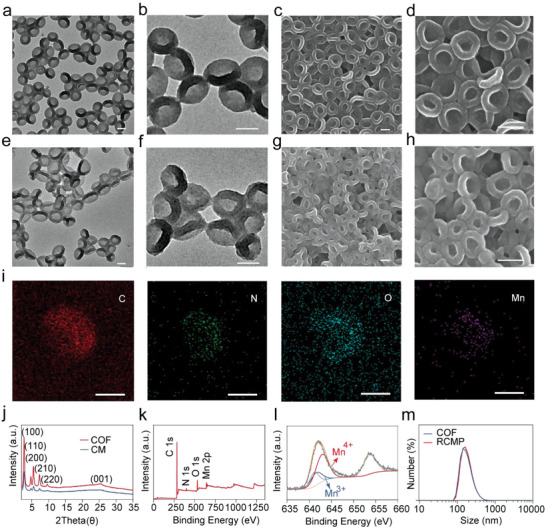
Characterization of pure and functionalized COF nanobowls. Transmission electron microscopy images of a,b) pure COF and e,f) COF‐MnO*_x_* nanobowls. Scanning electron microscopy images of c,d) COF and g,h) COF‐MnO*_x_* nanobowls. i) Elemental mapping images of COF‐MnO*_x_* nanobowls (CM). Scale bars: 100 nm. j) X‐ray powder diffraction patterns of COF and COF‐MnO*_x_* nanobowls. k) X‐ray photoelectron spectroscopy spectrum of COF‐MnO*_x_* nanobowls and l) high‐resolution spectrum of Mn 2p in COF‐MnO*_x_*. m) Size distribution of COF and functionalized COF nanobowls measured by dynamic light scattering. COF: covalent organic frameworks.

To fabricate the COF nanobowls, core–shell structured COFs were prepared by a typical dual‐ligand strategy using 1,3,5‐tris(4‐aminophenyl)benzene (TAPB) and 2,5‐dimethoxyterephthalaldehyde (DMTP) as the monomers and SiO_2_ nanospheres as the template (Figure S1, Supporting Information). The adjustable thickness of the COF layer made it possible to manipulate the shape of the COF in a controlled manner. When the COF layer was thick, a conventional hollow structure was obtained after etching the dense SiO_2_ core (Figure S2, Supporting Information). In contrast, COF nanobowls with a red blood cell‐like morphology can be fabricated with thinner shells. Transmission electron microscopy (TEM) and scanning electron microscopy images showed that the as‐synthesized COF nanobowls possessed a uniform morphology with an average particle size of ≈149 nm (**Figure** **1**a–d). MnO*_x_* covered the surface of the COF nanobowls via a redox reaction. The obtained COF‐MnO*_x_* featured nearly identical size and bowl‐shaped morphology as those of pure COF nanobowls, and highly dispersed nanoarchitectures were observed (Figure 1e–h). To verify the successful growth of MnO*_x_*, energy‐dispersive X‐ray elemental mapping was conducted to inspect the chemical elements in the COF‐MnO*_x_*. These results corroborated the presence of all the expected elements in COF‐MnO*_x_*, including C, N, O, and Mn (Figure 1i). Moreover, the X‐ray diffraction pattern illustrated the periodic crystalline frameworks of the COF nanobowls with high crystallinity (Figure 1j). Strong diffraction peaks at 2.72°, 4.81°, 5.63°, 7.39°, and 9.65°, corresponding to the (100), (110), (200), (210), and (220) planes of TAPB‐DMTP‐COF, respectively, appeared on both the COF nanobowls and COF‐MnO*_x_*. This is presumably because the polydopamine‐mediated in situ reaction can protect the crystalline frameworks of COF nanobowls from destruction induced by the strong oxidant KMnO_4_ (Figure S3, Supporting Information). Therefore, unconventional bowl‐shaped COF nanobowls with high crystallinity can be fabricated through precise control of the shell thickness and in situ redox reaction.

The valence state of Mn was investigated by X‐ray photoelectron spectroscopy. High‐resolution X‐ray photoelectron spectroscopy indicated the coexistence of Mn^3+^ and Mn^4+^ in COF‐MnO*_x_*, which is consistent with the manganese oxide reported in previous reports (Figure 1k,l).^[^ ^20^ ^]^ The formed MnO*_x_* is expected to play a dual role in blocking the sonodynamic activity of nanosensitizers and the catalytic effects of the nanozymes.^[^ ^21^ ^]^ To implement biological applications, PEG was modified onto the surface of COF‐MnO*_x_* to improve its physiological stability. Dynamic light scattering measurements revealed that the size distribution of COF nanobowls underwent slight changes during the fabrication process of PEGylated COF‐MnO*_x_* (Figure 1m). Moreover, the PEGylated COF‐MnO*_x_* showed no obvious change in average size in deionized water, phosphate‐buffered saline, or cell culture medium for 1, 3, 5, and 7 days (Figure S4, Supporting Information). The zeta potential results also demonstrated the slightly negative surface charge of PEGylated COF‐MnO*_x_*. The favorable colloidal stability of PEGylated COF‐MnO*_x_* could facilitate its application in biological environments (Figure S5, Supporting Information).

### 2.2 GSH‐Triggered Sonodynamic Process of Activatable Nanosensitizers

**Figure 2:**
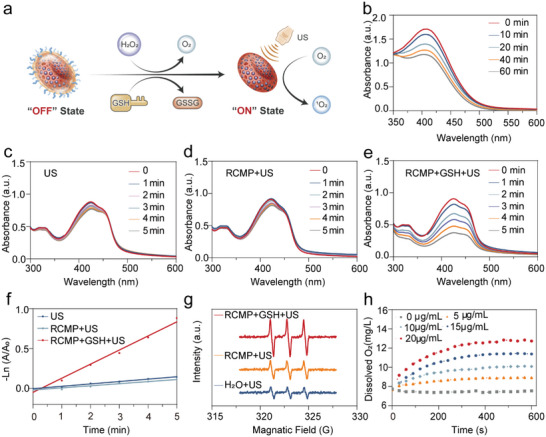
GSH‐activated sonodynamic effects of activatable nanosensitizers. a) Illustration of the GSH‐triggered sonodynamic process. b) Time‐dependent GSH depletion by RCMP. UV–vis spectra of DPBF solution containing c) only water, d) RCMP, and e) RCMP+GSH upon exposure to US irradiation (1 W cm^−2^, 1.0 MHz, 50% duty cycle) for different durations (0, 1, 2, 3, 4, and 5 min). f) Regression analysis of DPBF oxidation efficiency of different treatments under US irradiation for 5 min. g) Electron spin resonance spectra of different samples with 2,2,6,6‐tetramethylpiperidine (TEMP) as the radical trap. h) O_2_ production by the reaction of different concentrations RCMP with 100 µm H_2_O_2_. RCMP: RB@COF‐MnO*_x_*‐PEG; GSH: glutathione; COF: covalent organic frameworks; RB: rose bengal; PEG: polyethylene glycol; DPBF: 1,3‐diphenylisobenzofuran.

The synthesized COF‐MnO*_x_* nanobowls combine the merits of the highly crystallized porous structure of COFs and the tumor microenvironment‐responsiveness of MnO*_x_*,^[^ ^11^ ^]^ which motivated us to explore the potential of COF‐MnO*_x_* as responsive nanovehicles to implement activatable SDT. RB was selected as a model sonosensitizer and encapsulated into the COF framework (Figures S6 and S7, Supporting Information). The RB loading content reached as high as 20.6% (Figure S8, Supporting Information), which might be attributed to the rich *π*‐conjugated structure of the COFs.^[^ ^22^ ^]^ The characteristic absorbance peak of RB at 506 nm remained steady for RB@COF, but disappeared after the in situ growth of MnO*_x_* on the surface (Figure S9, Supporting Information). It is expected that the quenching effect of MnO*_x_* can effectively inactivate the photosensitivity of RB in tumor‐specific SDT. A similar mechanism has been demonstrated in previous studies.^[^ ^23^ ^]^ Due to its broad absorption, the outer MnO*_x_* could isolate the loaded photosensitizers from the light illumination to inhibit the photo‐triggered ROS generation. The photosensitizers could be activated upon the decomposition of MnO*_x_* layer and contact with O_2_, resulting in recovered photosensitizing capability. In addition, versatile responsive nanoplatforms have been developed previously based on the specific reaction between GSH and MnO*_x_*.^[^ ^24^ ^]^ However, the “blocking effect” of MnO*_x_* on the sonosensitization process has not been explored. It is important to inspect whether MnO*_x_* could block the US irradiation to activate the small‐molecule sonosensitizers. Therefore, we surmised that the outer MnO*_x_* would make the RCMP an activatable sonosensitizer to expose the encapsulated RB under high concentrations of GSH (≈10 mm) in cancer cells, leading to the activation of SDT (**Figure** **2**a).

Next, the GSH‐triggered sonodynamic effect of RCMP was investigated. GSH was used as a tumor‐specific inducer to study the transformability of RCMP. Specifically, RCMP was incubated with GSH for different durations, and GSH content was detected using the probe 5,5′‐dithiobis‐(2‐nitrobenzoic acid) (DTNB). The characteristic absorbance of DTNB decreased as the reaction time increased (Figure 2b), indicating that GSH could be depleted by RCMP. This is presumably due to the fact that the MnO*_x_* on the surface of RCMP can react with GSH, leading to the decomposition of MnO*_x_* and the reduction of GSH to GSSG. The energy dispersive X‐ray elemental mapping of RCMP demonstrated that the surface MnO*_x_* was completely consumed after reaction with 10 mm GSH (Figure S10, Supporting Information), which corresponded to the concentrations detected by inductively coupled plasma optical emission spectrometry (ICP‐OES) (Figure S11, Supporting Information). Owing to the specific redox reaction between MnO*_x_* and GSH, the decomposition of the MnO*_x_* layer can ensure a controlled therapeutic effect within the tumor cells, thus avoiding side effects. Furthermore, the sonodynamic activity of RCMP was investigated in the absence or presence of GSH, using 1,3‐diphenylisobenzofuran (DPBF) as a singlet oxygen (^1^O_2_) probe. Notably, the RCMP group exhibited a negligible change in absorbance at 410 nm under US irradiation, which was identical to that of the control group (DPBF+US) (Figure 2c,d). In sharp contrast, the characteristic absorption of DPBF markedly decreased with an increase in US exposure time when RCMP was pretreated with GSH (Figure 2e). Quantitative analysis also indicated that RCMP possessed significantly enhanced sonodynamic efficiency in the presence of GSH (Figure 2f). These results confirm that GSH can efficiently recuperate the suppressed sonodynamic efficiency of RCMP.

To further clarify the activatable sonodynamic behavior, the electron spin resonance spectrum was employed to ascertain the generation of ^1^O_2_ (Figure 2g). Analogously, under the same US exposure time, the activated nanosensitizers (RCMP+GSH) showed more intense signals of ^1^O_2_ (an intensity ratio of 1:1:1) than the prohibited group (RCMP) did, indicating GSH‐triggered sonodynamic efficacy. It is worth noting that RCMP can be more effectively activated under high‐concentration GSH (Figure S12, Supporting Information). Collectively, these findings suggest that RCMP can serve as an activatable nanosensitizer capable of switching from “off” to “on” states under GSH activation, exerting the sonodynamic therapeutic function. As GSH is one of the most abundant intracellular reductive species, the GSH depletion ability of RCMP might further boost its therapeutic efficacy. The catalase‐mimicking capability of MnO*_x_* has been extensively demonstrated; similarly, RCMP exhibited the ability to catalyze H_2_O_2_ to generate O_2_ to improve the local oxygen concentration (Figure 2h), favoring the oxygen‐dependent ROS generation of SDT. Overall, the above results demonstrate that RCMP can not only realize tumor‐specific activatable SDT to minimize undesirable side effects, but also combines the merits of nanozymes to profoundly augment the SDT efficiency by disrupting the intracellular redox homeostasis of cancer cells.

### 2.3 In Vitro Activatable SDT Enabled by Activatable Nanosensitizers

**Figure 3:**
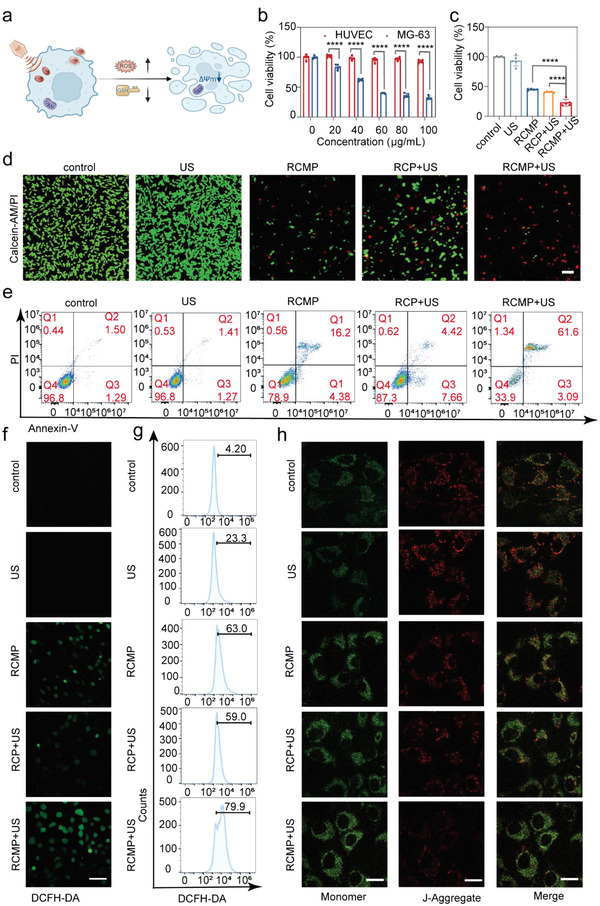
Enhanced in vitro therapeutic effects of activated SDT enabled by RCMP. a) Schematic illustration of RCMP‐mediated activated SDT to induce cell death. b) Relative cell viability of human umbilical vein endothelial cells and MG‐63 cells after incubation with various concentrations of RCMP (*n* = 5, mean ± s.d.). Statistical significance was calculated by two‐way ANOVA. c) Relative cell viability of MG‐63 cells after different treatments (*n* = 5, mean ± s.d.). Statistical significance was calculated by one‐way ANOVA. d) Confocal laser scanning microscopy images of MG‐63 cells stained with calcein acetoxymethyl ester (Calcein‐AM) and propidium iodide (PI) after various treatments. The scale bar is 100 µm. e) Flow cytometry analysis of MG‐63 cells stained by Annexin‐FITC and PI after various treatments. f) Intracellular ROS level of MG‐63 cells after different treatments. Scale bar: 100 µm. g) Flow cytometry of intracellular ROS level of MG‐63 cells after various treatments. h) Detection of mitochondrial membrane potential (ΔΨ) using JC‐1. Scale bar: 20 µm. Note: *p < 0.05, **p < 0.01, and ***p < 0.001, ****p < 0.001. SDT: sonodynamic therapy; RCMP: RB@COF‐MnO*_x_*‐PEG; GSH: glutathione; COF: covalent organic frameworks; RB: rose bengal; PEG: polyethylene glycol; ROS: reactive oxygen species.

The enhanced ROS generation efficiency and GSH depletion capability of RCMP guarantees its therapeutic efficiency in cancer cells. Logically, upon US irradiation, RCMP can efficiently initiate cancer cell apoptosis, which is a major mechanism of programmed cell death. In addition, GSH depletion is involved in cell ferroptosis via the inhibition of GPX4. Hence, a series of in vitro cell experiments was performed to explore the feasibility of the activatable nanosensitizers for in vitro ferroptosis‐augmented SDT (**Figure** **3**a).

First, the cell‐killing capability of RCMP was determined using the cell‐counting kit‐8 (CCK‐8) assay. After co‐incubation for 24 h, RCMP showed remarkable dose‐dependent killing effects on human osteosarcoma cells (MG‐63) but negligible cytotoxicity in human umbilical vein endothelial cells (HUVEC) (Figure 3b). The tumor‐specific cytotoxicity is presumably owing to higher endogenous GSH levels in cancer cells than in normal cells. The synergistic therapeutic efficacy was further evaluated using MG‐63 cells and five groups were set as follows: cells treated with complete culture medium as the control group, US group, RCMP group, conventional SDT group of RCP (RCP represents RB‐loaded COF nanobowls without the growth of MnO*_x_* layer) plus US irradiation, and GSH‐activated SDT group of RCMP plus US irradiation. Compared to the other groups, the GSH‐activated SDT group exhibited the highest degree of toxicity to MG‐63 cells (Figure 3c). The cell‐killing effect of RCMP was significantly stronger than that of the MnO*_x_*‐free RCP group under the same conditions of US irradiation. This might be explained by the GSH‐depletion and oxygen evolution capabilities of RCMP, as demonstrated above, realizing activatable SDT to boost therapeutic efficacy. The live–dead staining images also revealed that more living cancer cells existed in the other groups than in the RCMP+US group (Figure 3d and Figure S13, Supporting Information), indicating the superior therapeutic efficacy of RCMP compared to that of other agents. Subsequently, the apoptosis of treated MG‐63 cells was quantitatively investigated by flow cytometry (Figure 3e). US irradiation alone resulted in imperceptible apoptosis, and the apoptosis rates of RCMP‐ and RCP‐treated cells were 21.1% and 12.7% (Table S1, Supporting Information), respectively. In contrast, treatment with RCMP followed by US irradiation dramatically enhanced MG‐63 cell apoptosis (≈66.1%). This tendency is consistent with the above results, which demonstrates that nanosensitizers with improved sonodynamic efficacy can realize favorable cell‐killing effects.

Furthermore, the enhanced sonodynamic effects of RCMP were visualized using fluorescent staining. A 2,7‐dichlorodihydrofluorescein diacetate (DCFH‐DA) probe was used to inspect intracellular ROS production levels in MG‐63 cells (Figure 3f). MG‐63 cells treated with the culture medium were used as negative controls. The green fluorescence from oxidized DCFH was weak in the US‐treated group and the control. Bright green fluorescence was observed in both the RCMP and RCP+US groups, suggesting elevated levels of ROS. The RCMP+US group exhibited considerably more fluorescence than the former two groups, which is consistent with the results of flow cytometry (Figure 3g). Moreover, the JC‐1 assay kit was employed to measure the changes in mitochondrial membrane potential of MG‐63 cells because the large accumulation of ROS in cells leads to mitochondrial membrane depolarization and subsequent apoptosis. The probe emits green and red fluorescence to indicate damaged mitochondria with a low potential and normal mitochondria with a high potential, respectively.^[^ ^25^ ^]^ Similarly, treatment with conventional SDT (RCP+US) or RCMP alone caused only a moderate loss of cellular mitochondrial membrane potential (Figure 3h), as evidenced by the increased green/red fluorescence in confocal laser scanning microscopy (CLSM) images. However, a large portion of red JC‐1 aggregates was observed in cells treated with RCMP+US, implying mitochondrial dysfunction induced by GSH‐activated SDT.

### 2.4 Mechanism of Activated and Ferroptosis‐Augmented SDT

**Figure 4:**
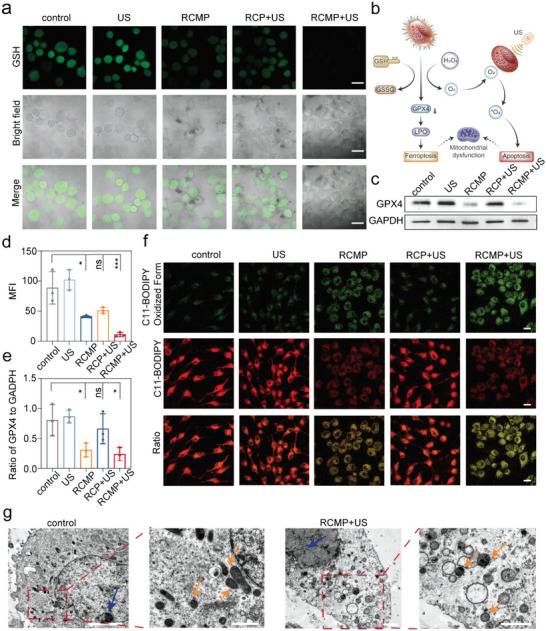
Ferroptosis mechanism of RCMP‐mediated SDT. a) Confocal images of MG‐63 cells stained with Thiol Tracker violet (GSH detection reagent). b) Schematic illustration of the proposed molecular mechanism for the killing of cancer cells based on ferroptosis or apoptosis induced by GSH‐activated SDT. c) Western blotting of GPX4 in MG‐63 cells. d) Fluorescent intensity corresponding to different groups in (a) that represents the intracellular GSH level (*n* = 3, mean ± s.d.) Statistical significance was calculated by one‐way ANOVA. e) Quantitative analysis of GPX4 protein ratio to GAPDH for each group (*n* = 3, mean ± s.d.) Statistical significance was calculated by one‐way ANOVA. f) Confocal laser scanning microscopy observation on the intracellular distribution of LPO indicated by BODIPY 581/591‐C11 in MG‐63 cells under various treatments. Scale bar: 20 µm. g) Bio‐transmission electron microscopy images of cells treated with cell medium (control) and RCMP+US irradiation (GSH‐activated SDT). Scale bar: 1 µm. Mitochondria and chromatin are indicated by orange and blue arrows, respectively. Note: *p < 0.05, **p < 0.01, ***p < 0.001, and ****p < 0.001. SDT: sonodynamic therapy; RCMP: RB@COF‐MnO*_x_*‐PEG; GSH: glutathione; COF: covalent organic frameworks; RB: rose bengal; PEG: polyethylene glycol; GPX4: glutathione peroxidase 4; LPO: lipid peroxides; US: ultrasound; GAPDH: glyceraldehyde‐3‐phosphate dehydrogenase.

To explore the detailed mechanism of GSH‐activated SDT, the GSH depletion capability of RCMP was studied further, considering that GSH plays a major role in the detoxification of intracellular ROS. The thiol‐tracker violet staining assay was used to detect intracellular GSH levels in cells under different treatments. The cells treated with RCMP alone exhibited weaker green fluorescence than the control and US groups did (**Figure** **4**a), indicating the GSH‐depletion capability of RCMP. The fluorescence intensity of cells treated with RCP+US also decreased markedly, which implies that conventional SDT could consume intracellular GSH by increasing the ROS level. Notably, the RCMP+US group exhibited the weakest fluorescence among these groups (Figure 4d). The significantly decreased GSH content in this group was attributed to the simultaneous ROS generation and GSH depletion effects of the activatable SDT.

Because the decrease in GSH levels directly impairs the anti‐oxidation ability of cancer cells and further causes the downregulation of GPX4, activated SDT is expected to induce both apoptosis and ferroptosis in cancer cells (Figure 4b). Thus, we next investigated the expression of GPX4 in MG‐63 cells under different treatments using western blotting with glyceraldehyde‐3‐phosphate dehydrogenase as an internal control. The GPX4 band of the RCMP+US group was much weaker than that of the other groups (Figure 4c,e), suggesting that the RCMP‐mediated activatable sonodynamic process effectively induced the downregulation of GPX4 in MG‐63 cells. This result is in accordance with the hypothesis that RCMP‐enabled tumor‐specific SDT can inactivate GPX4 in MG‐63 cells through bilaterally enhanced ROS generation and GSH depletion.^[^ ^26^ ^]^ In addition, GPX4 is an essential factor in counteracting LPO and maintaining membrane lipid bilayer homeostasis. The inactivation of GPX4 and depletion of GSH might give rise to the intracellular accumulation of LPO, a significant biomarker of ferroptosis.^[^ ^27^ ^]^ BODIPY581/591‐C11, an LPO‐sensitive probe, was used to detect LPO levels in MG‐63 cells after various treatments. The probe exhibited spectral separation between the non‐oxidized (595 nm) and oxidized (520 nm) forms. The cells treated with RCMP+US presented the most intense green fluorescence but the weakest red fluorescence (Figure 4f), revealing that RCMP could produce massive LPO under US irradiation and lead to ferroptosis. To investigate the specific morphological changes owing to ferroptosis, MG‐63 cells subjected to activatable SDT were observed using bio‐TEM (Figure 4g and Figure S14, Supporting Information). Compared with normal cells, disintegrated cell membranes with effervescence and normal nuclear morphology, but lacking chromatin condensation, were observed in the experimental samples. The overall number of mitochondria in the cells treated with RCMP+US also decreased, accompanied by breakage, dissolution, and disappearance of mitochondrial ridges and an increase in mitochondrial membrane density. These microscopic morphological changes were consistent with the features of ferroptosis. Therefore, upon US irradiation, RCMP results in cancer cell ferroptosis, synergistically improving sonodynamic therapeutic efficacy.

**Figure 5:**
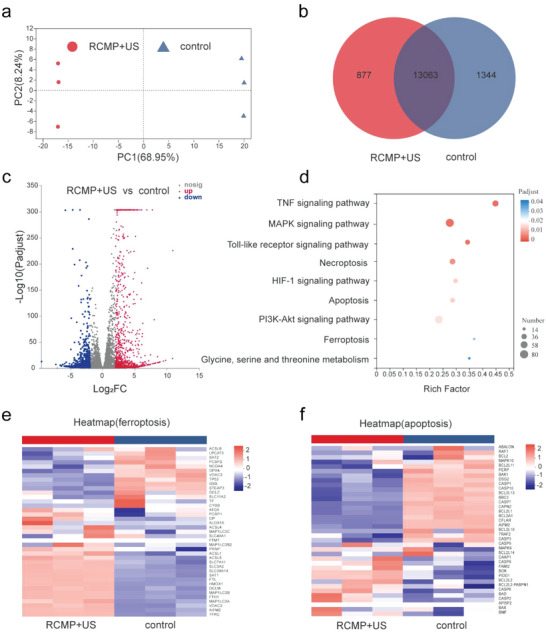
Biological mechanisms of RCMP under ultrasound irradiation by RNA sequencing. a) Principal component analysis and b) Venn diagram of the differentially expressed genes in the control and RCMP+US irradiation groups. c) Volcano plot of the differentially expressed genes in the control and RCMP+US irradiation groups. d) Bubble diagram of the differentially expressed genes enriched in the Kyoto Encyclopedia of Genes and Genomes (KEGG) pathway. e,f) Heat map of genes that were differentially expressed in the MG‐63 cells treated RCMP upon US irradiation as compared to the control (fold change [FC] ≥ 2.0 [or −2.0], *p* < 0.05). RCMP: RB@COF‐MnO*_x_*‐PEG; COF: covalent organic frameworks; RB: rose bengal; PEG: polyethylene glycol; US: ultrasound.

Transcriptomic analysis was used to further study the underlying mechanisms of ferroptosis‐augmented SDT. MG‐63 cells treated with phosphate‐buffered saline ( or RCMP+US were collected and subjected to RNA sequencing. Principal component analysis revealed significant differences in gene expression between the control and RCMP+US groups, which was also evidenced by the results obtained from the heat map analysis (**Figure** **5**a and Figure S15, Supporting Information). As indicated in the Venn diagram, 13 063 genes were co‐expressed in the two groups of samples, whereas 877 genes were expressed in only the RCMP+US irradiation group (Figure 5b). Volcano plots exhibited differentially expressed genes (fold change [FC] ≥ 2.0 [or −2.0], *p* < 0.05) (Figure 5c). The significant differences in gene expression suggest that RCMP, upon US irradiation, has a distinct effect on the molecular biological mechanisms of MG‐63 cells. To identify the pathways involved in RCMP‐induced ferroptosis and SDT, Kyoto Encyclopedia of Genes and Genomes analysis was performed to uncover gene set enrichment (Figure 5d). Multiple cellular pathways including tumor necrosis factor, mitogen‐activated protein kinase (MAPK), apoptosis, and ferroptosis signaling pathways were significantly enriched after treatment with RCMP‐enabled activatable SDT. Among these pathways, the MAPK signaling pathway can be activated by elevated intracellular ROS to induce apoptosis.^[^ ^28^ ^]^ In addition, tumor necrosis factor is involved in the caspase‐8‐mediated apoptotic pathway, which also induces apoptosis.^[^ ^29^ ^]^ The well‐known ferroptosis‐related genes, including GPX4, SLC7A11, SLC9A14, and TFRC, were distinctly regulated (Figure 5e), suggesting the occurrence of ferroptosis.^[^ ^30^ ^]^ In addition, treatment with RCMP+US significantly upregulated or downregulated the expression of apoptosis‐related genes in tumor cells, such as fatty acid synthase, tumor necrosis factor, and caspase, which are closely related to apoptosis (Figure 5f).^[^ ^31^ ^]^ Collectively, upon US irradiation, RCMP could actively induce apoptosis and ferroptosis in cancer cells, guaranteeing highly efficient cell death under activatable SDT.

### 2.5 In Vivo Biosafety of RCMP

To evaluate the biocompatibility and biosafety of RCMP, healthy female ICR mice were intravenously injected with RCMP (10 mg kg^−1^) on days 7, 14, and 21 prior to execution, and control mice were administered saline. Blood biochemical examination showed that RCMP had negligible effects on liver and kidney function indicators, including alanine aminotransferase, aspartate aminotransferase, alkaline phosphatase, blood urea nitrogen, and creatinine, suggesting that RCMP has no significant effect on normal liver and kidney function in either the long‐ or short‐term (Figure S16, Supporting Information). Blood indices were found to be within the normal reference range according to routine blood tests (Figure S17, Supporting Information), which demonstrates the hemocompatibility of RCMP. In addition, the major organs (heart, liver, spleen, lung, and kidney) of the treated mice were harvested for hematoxylin and eosin (H&E) staining (Figure S18, Supporting Information). No significant injury or obvious inflammation was observed from histopathological analyses after the injection of RCMP, further demonstrating its favorable histocompatibility. The high biosafety of RCMP permits further in vivo application.

### 2.6 In Vivo Evaluation of Ferroptosis‐Augmented SDT on Osteosarcoma

**Figure 6:**
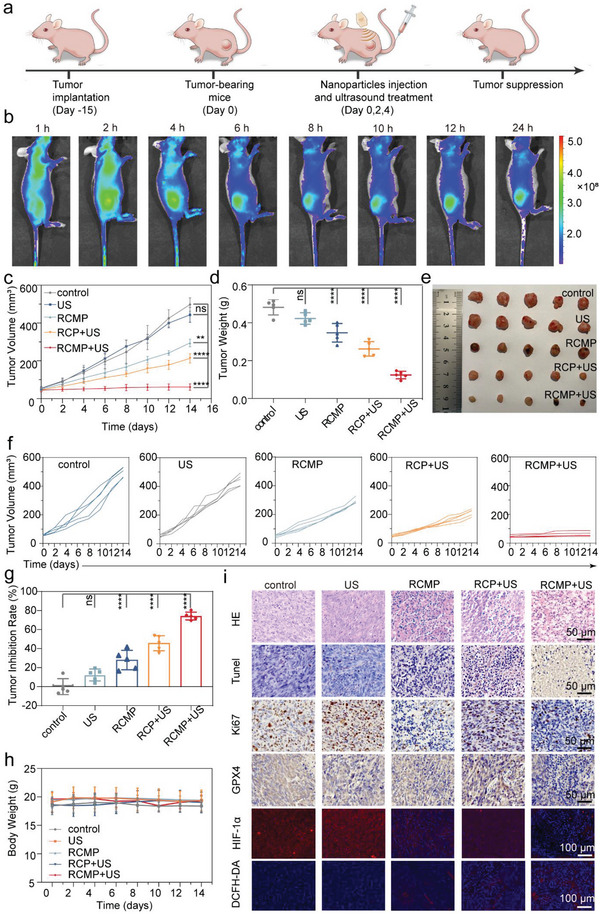
In vivo antitumor efficacy of RCMP‐mediated ferroptosis‐augmented SDT. a) Schematic illustration of in vivo therapeutic protocols. b) In vivo fluorescence images of cy5.5 in MNNG‐HOS tumor‐bearing mice after the intravenous injection of RCMP‐cy5.5. c) Tumor volume changes in the mice receiving indicated treatments every 2 days (*n* = 5, mean ± s.d.) Statistical significance was calculated by two‐way ANOVA. d) Average weight and e) photographs of tumors harvested from tumor‐bearing nude mice in different groups after the 14‐day treatment period (*n* = 5 in each group, mean ± s.d.) Statistical significance was calculated by one‐way ANOVA. f) The growth curve of tumor volume of each mouse during the therapeutic period. g) Calculated tumor‐inhibition rate of different groups after the treatments (*n* = 5, mean ± s.d.). Statistical significance was calculated by one‐way ANOVA. h) Body weight changes in mice in each group every 2 days (*n* = 5, mean ± s.d.). i) Histological images of tumor slices stained with H&E, TUNEL, antigen Ki‐67, GPX4, HIF‐1*α*, and DCFH‐DA collected from the tumor‐bearing nude mice at day 14. Note: *p < 0.05, **p < 0.01, ***p < 0.001, ****p < 0.001. SDT: sonodynamic therapy; RCMP: RB@COF‐MnO*_x_*‐PEG; COF: covalent organic frameworks; RB: rose bengal; PEG: polyethylene glycol; GPX4: glutathione peroxidase 4; US: ultrasound; H&E: hematoxylin and eosin; TUNEL: TdT‐mediated dUTP nick‐end labeling; HIF‐1 *α*: hypoxia‐inducible factor‐1*α*; DCFH‐DA: 2,7‐dichlorodihydrofluorescein diacetate.

The desirable in vitro therapeutic performance and in vivo biosafety of RCMP encouraged us to further evaluate its antitumor efficacy in vivo, using the MNNG‐HOS xenograft osteosarcoma model (**Figure** **6**a). The biological effects of the activatable nanosensitizers were preliminarily studied because bowl‐shaped COFs have not been used in biomedicine. Given the distinctive nanoscale morphology, tumor accumulation of the developed nanosensitizers was assessed by fluorescent imaging. Specifically, RCMP with bowl‐shaped morphology and its spherical counterpart SiO_2_@RCMP without the etching of SiO_2_ were labeled with near‐infrared dye cy5.5, and intravenously injected into tumor‐bearing mice. The captured fluorescent images and the corresponding quantitation revealed that RCMP could effectively accumulate at the tumor site and be retained for a long period (Figure 6b and Figures S19 and S20, Supporting Information). It is worth noting that RCMP also exhibits higher fluorescence intensity and longer retention time than un‐etched SiO_2_@RCMP, implying its superior tumor accumulation efficiency. As previously reported, tuning the elasticity of nanoparticles holds potential to improve their delivery efficiency to tumors. The unique bowl‐like shape of RCMP with reduced stiffness is speculated to contribute to improved tumor accumulation and retention.^[^ ^32^ ^]^ Additionally, it is observed that the cell internalization of RCMP outperforms that of the spherical SiO_2_@RCMP with higher stiffness (Figures S21 and S22, Supporting Information), which further validates the exceptional biological effects of bowl‐shaped nanosensitizers. It could be deduced that the unconventional morphology and appropriate stiffness of RCMP might facilitate its cellular phagocytosis, which remains to be investigated in detail in future.^[^ ^33^ ^]^ Although the underlying structure–performance relationship requires in‐depth investigation in the future, the enhanced nano‐bio interaction by regulating the morphology of nanoscale COFs may provide insight into the development of COF‐based nanomedicine with distinctive nanostructures and high performance.

Given the synergistically enhanced sonodynamic efficacy and favorable biological effects, the therapeutic efficiency of RCMP was investigated. Female BALB/c nude mice bearing MNNG‐HOS tumors were randomly divided into five groups (*n* = 5) for different treatments when the tumor volume reached ≈50 mm^3^: saline (control), US (1 MHz, 50% duty cycle, 1 W cm^−2^), only RCMP, RCP+US, and RCMP+US (10 mg kg^−1^) groups. According to the tumor volume curve (Figure 6c), the RCMP and RCP+US groups exhibited moderate inhibition of tumor growth compared to the control group, whereas relatively potent inhibition of tumor growth was observed in the RCMP+US group. Moreover, upon US irradiation, the tumor suppression efficacy of RCMP was superior to that of RB‐COF throughout the 2‐week observation period. At the end of the treatment, the excised tumors were weighed and photographed, and the results were positively correlated with the tumor volumes of the mice (Figure 6d–f and Figure S23, Supporting Information). Based on the above data, the growth inhibition rate of the tumors was calculated (Figure 6g). A high inhibition rate of 74% was achieved in the RCMP+US group compared to the RCMP (30%) and RCP+US (46%) groups, suggesting the superior antitumor efficacy of RCMP upon US irradiation. In addition, normal fluctuations were observed in all groups during the continuous monitoring period, and H&E staining of the major organs of the mice in each group showed no tissue damage at the end of the treatment. The results demonstrated that all treatments were biocompatible and well‐tolerated (Figure 6h and Figure S24, Supporting Information).

The therapeutic performance of each group was further analyzed by H&E staining; immunohistochemical staining with TdT‐mediated dUTP nick‐end labeling (TUNEL), Ki‐67, and GPX4; and immunofluorescence staining with DCFH‐DA and hypoxia‐inducible factor (HIF)‐1*α* (Figure 6i). The H&E staining revealed bulky cavities and a large amount of overflowing cytoplasm in the tumor tissue of the RCMP+US group, indicating apoptosis or necrosis of tumor cells. Treatment with RCMP+US resulted in massive apoptosis (elevated percentage of TUNEL positivity). Furthermore, the immunohistochemical positivity rate of Ki‐67 was the lowest in the RCMP+US group, further confirming the significant inhibitory effect on tumor cell proliferation. Notably, GPX4 expression was remarkably decreased in the tumor tissues of the RCMP+US group, demonstrating that the favorable antitumor efficacy of RCMP could be ascribed to ferroptosis‐augmented SDT. Moreover, the decreased expression of HIF‐1*α* in RCMP and RCMP+US groups suggests that the hypoxia of tumors can be ameliorated to some extent due to the catalase‐mimicking ability of MnO*_x_*. Accordingly, the DCFH‐DA staining results evidence that the ROS production of RCMP+US group is stronger than that of RCP + US group. Therefore, in vivo results corroborated that the developed activatable nanosensitizer, RCMP, could achieve high antitumor efficacy under US irradiation by synergistically enhancing and ferroptosis‐augmenting SDT.

## 3 Conclusion

In summary, we successfully synthesized a COF nanobowl‐based nanosensitizer with a distinctive nanoscale morphology and activatable sonodynamic activity. COF nanobowls feature high crystallinity and abundant porosity, which permit efficient loading of the small‐molecule sonosensitizer RB. Activatable nanosensitizers were obtained by engineering the RB‐loaded COF nanobowls with MnO*_x_* shells. Benefiting from the GSH‐responsive degradation behavior, MnO*_x_* serves as a detachable “gatekeeper” to block the sonodynamic effects of the RCMP. The tumor‐specific nanosensitizer switched from the “off” to “on” state to exert therapeutic efficacy at the tumor site under US irradiation, enabling the GSH‐activated sonodynamic process and concomitantly inducing downregulation of GPX4. In vitro experiments revealed that RCMP combined with US irradiation could disrupt intracellular redox hemostasis and cause ferroptosis in cancer cells. These synergistic effects amplified therapeutic efficacy. In vivo evaluations also validated that the bowl‐shaped morphology rendered the COF nanosensitizers exceptional enhancement effects on tumor accumulation and retention. Consequently, RCMP exhibited high efficacy in inhibiting tumor growth under US irradiation. Overall, this study paves the way for the controlled synthesis and development of COF‐based nanosensitizers with unconventional morphology, offering a new strategy to realize activatable and ferroptosis‐augmented SDT as well as extending the biomedical application of nanoscale COFs.

## 4 Experimental Section

### Materials

Tetraethyl orthosilicate, polyvinylpyrrolidone (PVP, Mw ≈ 40 000), and polyethyleneimine (PEI, Mw ≈ 10 000), GSH, and DTNB were purchased from Aladdin Chemistry, Co., Ltd. Methanol, dopamine, and tris‐(hydroxymethyl)‐aminomethane (Tris), TAPB were purchased from Shanghai Macklin Biochemical Co., Ltd. DPBF was bought from Adamas Reagent, Ltd. DMTP was bought from Sigma‐Aldrich. Potassium permanganate (KMnO_4_), absolute ethanol, and sodium hydroxide (NaOH) were obtained from Sinopharm Chemical Reagent Co., Ltd. CCK‐8 was bought from Yeasen Biotechnology (Shanghai) Co., Ltd. DCFH‐DA probe and Annexin V‐FITC and PI dual staining kit were obtained from Beyotime Biotechnology. BODIPY 581/591‐C11 was purchased from Med. Chem. Express (New Jersey, USA). Calcein‐AM/PI double stain kit was obtained from Dojindo Molecular Technologies.

### Synthesis of SiO_2_@COF and Selectively Etching of SiO_2_

Specifically, 2.0 mL of PEI/PVP‐modified SiO_2_ was centrifuged and dispersed into 20.0 mL of anhydrous acetonitrile containing 200 mg of PVP. The dispersion was treated with ultrasonic for more than 0.5 h. Then, 5 mg of DMTP and 6 mg of TAPB were added to the above dispersion. After stirring for 5 min, 200 µL of glacial acetic acid was further added. The reaction was maintained at room temperature for 4 h. Afterward, 800 µL of acetic acid was further added and the mixture was heated to 80 °C for another 12 h reaction. The products were obtained and washed by centrifugation. To obtain bowl‐shaped COF, the SiO_2_@COF were dispersed in 2 m NaOH aqueous solution and stirred overnight.

### Preparation of RCMP

To synthesize RB@COF, 2 mg of RB were added into 5 mL dispersion of COF and stirred for 12 h. The products were collected and washed by high‐speed centrifugation. Then, dopamine and Tris were added to make the concentration of dopamine of 0.25 mg mL^−1^ and Tris of 1 mg mL^−1^ in the mixture, respectively, and then stirred lasted for 12 h. The solution was taken out for further centrifugation and washing. Finally, the products were added with 200 µL KMnO_4_ solution (1 mg mL^−1^), stirring for another 0.5 h. Finally, the obtained RCM particles were mixed with 10.0 mg of amino‐terminated polyethylene glycol (PEG‐NH_2_, M_w_ ≈ 2000) in water and stirred overnight. The obtained RCMP was collected, washed, and dispersed in water for further use. The nanosensitizers without MnO*_x_* (RCP) and SiO_2_@RCMP were also prepared by the same procedures to RCMP, except that the growth of MnO*_x_* and the etching of SiO_2_ were not performed. Besides, the KMnO_4_ solution was added into COF nanobowls to allow direct redox reaction without the assistance of polydopamine, obtaining MnO*_x_*‐coated COF nanobowls for comparison.

### Characterization

TEM images were observed via JOEL‐2100f at an acceleration voltage of 200 kV, which was equipped with energy dispersion spectrum (EDS). X‐ray photoelectron spectroscopy (Thermo Scientific K‐Alpha) analysis was performed through K‐Alpha to determine the elemental types and valence states of the Mn component of RB@MnO*_x_*‐COF. X‐ray powder diffraction patterns were scanned from 2° to 30° on a Rigaku automated X‐ray diffractometer (Smartlab, Rigaku Co. Ltd., Tokyo, Japan). The hydrodynamic size and zeta potential of different nanoparticles were detected by dynamic light scattering (Zetasizer Nano ZSE). The UV‐vis  absorption spectra were recorded by UV‐1800 spectrometer (MAPADA, China). Electron spin resonance spectrum was obtained by JEOL‐FA200 ESR spectrometer (JEOL Company Ltd., Japan). CLSM (CLSM 710; Carl Zeiss, Germany) was applied for acquiring the confocal images. The absorbance for CCK‐8 assay was recorded at a wavelength of 450 nm with a Tecan's Infinite M200 microplate reader. Flow cytometry (Beckman, USA) was used for analyzing cell apoptosis and the intracellular ROS generation. US therapy instruments (Encore Medical International, Inc.) was applied as an instrument to perform SDT. ICP‐OES (Agilent Technologies, USA) was conducted for quantitative analysis of the content of Mn element.

### Glutathione Consumption

GSH depletion was detected by UV–vis spectroscopy. RCMP (50 µg/mL) was mixed with GSH (10 mm) at 37°C. After different durations of reaction, 50 µL of DTNB (1.5 mg/mL) was added to the solution and reacted for another 10 min. After centrifugation to remove RCMP from the solution, the absorbance of the supernatant was measured.

### Evaluation of ^1^O_2_ Generation In Vitro

150 µL RCMP solution (1 mg mL^−1^) was mixed with 150 µL GSH (20 mM) so that the final concentration of GSH in the reaction solution was 10 mm. The duration of reaction was 30 min. 40 µL of DPBF (1 mg mL^−1^) was added to the 2.96 mL diluent of 1) RCMP (50 µg mL^−1^) solution, or 2) a mixture of RCMP (50 µg mL^−1^) and GSH. The US irradiation (1.0 W cm^−2^, 1.0 MHz, 50% duty cycle) was performed for 5 min. UV–vis absorption spectra of the above solutions were recorded every minute. Furthermore, different concentrations of GSH solution were applied to deplete the surface MnO*_x_* of RCMP. The mixture of RCMP and GSH was pre‐reacted for 10 min, and the mixture was centrifuged to remove the redundant GSH. Then the DPBF was applied to evaluate the sonodynamic efficiency of pretreated‐RCMP. The duration and power of US were consistent with above experiment. The UV–vis absorption spectra were recorded every minute as well. Besides, Electron spin resonance spectroscopy measurement was conducted to monitor the ^1^O_2_ generation with TEMP as a trapping agent.

### In Vitro Oxygen Production

RCMP solutions at different concentrations (0, 5, 15, 20, 25 µg mL^−1^) were reacted with 100 µm H_2_O_2_ solution at room temperature, and the dissolved oxygen content in the solution was monitored in real time, and the dissolved oxygen content displayed on the dissolved oxygen meter was recorded every 30 s.

### Cell Culture

Human osteosarcoma cell lines (MG‐63 cells and HOS‐MNNG cells) and HUVEC were purchased from Shanghai Institute of Cell Science, China. MG‐63 cells were cultured in MEM medium (Gbico) containing 10% fetal bovine serum (FBS, Gbico) and 1% penicillin–streptomycin (Invitrogen) at temperature of 37 °C in an incubator containing 5% CO_2_.

HOS‐MNNG cells and HUVEC cells were cultured in DMEM medium (Gbico) containing 10% FBS (Gbico) and 1% penicillin–streptomycin (Invitrogen) in an incubator at 37 °C with 5% CO_2_.

### In Vitro Cell Viability Evaluation

To investigate the cytotoxicity of RCMP+US, HUVEC and MG‐63 cells were cultured overnight on 96‐well plates. Then, cells were treated with different concentrations of RCMP and incubated for 24 h. After different treatments, cell viability was determined by the CCK‐8 assay. The absorbance of each well was measured with a microplate reader, and the ratio of the absorbance of each well at 450 nm to that of the untreated control was calculated to obtain the relative cell viability. After selecting an appropriate concentration based on the above results, the wells were incubated with different nanomaterials, and some groups were irradiated with US (1.0 W cm^−2^, 1.0 MHz, 5 min, 50% duty cycle). The absorbance of each well at 450 nm was measured again by the microplate reader and the relative cell viability was calculated.

### In Vitro Live–Dead Staining

MG‐63 cells were inoculated in confocal dishes and cultured overnight. MG‐63 cells were subjected to different treatments, including PBS, US irradiation, RCMP (60 µg mL^−1^), RCP (60 µg mL^−1^) + US irradiation, and RCMP (60 µg mL^−1^) + US irradiation (1.0 W cm^−2^, 1.0 MHz, 5 min, 50% duty cycle). Live and dead cells were labeled with Calcein‐AM (Ex/Em = 488/530 nm) and PI (Ex/Em = 535/617 nm), respectively. After co‐incubation for 30 min, cells were washed twice with PBS and subsequently observed by CLSM.

### In Vitro Annexin V‐FITC/PI Apoptosis Assay

MG‐63 cells were inoculated in 6‐well plates and cultured overnight. MG‐63 cells were treated with different treatments, including PBS, US irradiation, RCMP (60 µg mL^−1^), RCP (60 µg mL^−1^) + US irradiation, and RCMP (60 µg mL^−1^) + US irradiation (1.0 W cm^−2^, 1.0 MHz, 5 min, 50% duty cycle), collected and washed two times repeatedly with PBS, and cells were resuspended with the binding solution. The cells were labeled with Annexin V‐FITC and PI and then incubated for 15 min at room temperature. Finally, the cell apoptosis caused by different treatment were analyzed by flow cytometry.

### Intracellular Reactive Oxygen Specials Evaluation

MG‐63 cells were inoculated in confocal dishes and cultured overnight. MG‐63 cells received different treatments, including PBS, US irradiation, RCMP (60 µg mL^−1^), RCP (60 µg mL^−1^) + US irradiation, and RCMP (60 µg mL^−1^) + US irradiation (1.0 W cm^−2^, 1.0 MHz, 5 min, 50% duty cycle). After washing with PBS, the cells were incubated with DCFH‐DA staining solution in the dark for 30 min at 37 °C. After loading the fluorescent probes, the cells were further subjected to US irradiation. Subsequently, the fluorescence intensity was observed by confocal microscopy.

To further quantitatively investigate the generation of ROS, cells were seeded in 6‐well plates. After different treatments, the cells were incubated with the dye for 30 min and then collected with 0.05% trypsin‐EDTA solution, suspended in fresh medium, and immediately analyzed by flow cytometry.

### Mitochondrial Membrane Potential Assay

MG‐63 cells were incubated in confocal dishes and treated separately. In brief, after washing several times with PBS, the JC‐1 staining working solution was added and the cells were incubated for 20 min at 37 °C in the incubator. A shift from JC‐1 monomer (Ex/Em = 515/529 nm) to JC‐1 aggregates (Ex/Em = 585/590 nm) occurred if there was a decrease in mitochondrial membrane potential. Intracellular fluorescence was observed by CLSM.

### Intracellular Glutathione Depletion Assay

MG‐63 cells were inoculated in confocal dishes for 24 h. The cells were incubated with PBS, US irradiation, RCMP (60 µg mL^−1^), RCP (60 µg mL^−1^) + US irradiation, and RCMP (60 µg mL^−1^) + US irradiation (1.0 W cm^−2^, 1.0 MHz, 5 min, 50% duty cycle). After various treatments, cells were stained with ThiolTracker Violet (10 µm) for 30 min. Cell samples were washed twice with PBS and observed on CLSM. Intracellular fluorescent signals were observed and quantified by ImageJ to assess the extent of GSH depletion.

### Western Blot

MG‐63 cells were inoculated in 6‐well plates at the desired density of cells and cultured for 12 h. Subsequently, the cells were treated with PBS, US irradiation, RCMP (60 µg mL^−1^), RCP (60 µg mL^−1^) + US irradiation, and RCMP (60 µg mL^−1^) + US irradiation (1.0 W cm^−2^, 1.0 MHz, 5 min, 50% duty cycle), respectively. After 24 h incubation, cells were rinsed with ice‐cold PBS, harvested, and fully lysed with lysis buffer on ice for 30–60 min. The supernatant was collected, and the protein in the supernatant was collected by centrifugation at 12000 rpm for 15 min. Protein concentrations were then determined by Omni‐Easy instant BCA protein assay kit (EpiZyme, China). Proteins were separated in a 4–20% gradient gel (EpiZyme, China) and transferred to polyvinylidene fluoride (IPVH00010, Millipore, USA) membranes. The membranes were transferred to the blocking buffers for 2 h, followed by rinsing with tris‐buffered saline‐Tween 20 buffer (TBS‐T). Then, they were incubated with primary antibody (GPX4, Abcam, USA) overnight at 4 °C. The target protein was incubated with a secondary antibody for 1 h at room temperature and then rinsed three times with TBS‐T. Finally, the protein bands were visualized with a Bio‐Rad ChemiDoc Imaging System (Bio‐Rad, China).

### Intracellular Lipid Peroxide Evaluation

MG‐63 cells were seeded in CLSM dishes and cultured overnight. When the cell density reached 80%, the sample cells were treated with PBS, US irradiation, RCMP (60 µg mL^−1^), RCP (60 µg mL^−1^) + US irradiation, and RCMP (60 µg mL^−1^) + US irradiation (1.0 W cm^−2^, 1.0 MHz, 5 min, 50% duty cycle), and incubated for another 12 h. At the end of the treatment, the cells were washed three times with PBS. The LPO probe BODIPY581/591‐C11 was configured into a working solution of 10 µm and co‐incubated with cells in an incubator at 37 °C for 30 min. After washing again with PBS, intracellular LPO was observed with CLSM. The excitation wavelength was 555 nm for the non‐oxidized form and 488 nm for the oxidized form.

### Biological TEM imaging

MG‐63 cells were incubated with PBS or RCMP+US (1.0 W cm^−2^, 1.0 MHz, 5 min, 50% duty cycle) for 24 h, respectively. The treated cells were collected, washed twice with PBS, and then added to 2.5% glutaraldehyde fixative at 4 °C overnight. The samples were rinsed three times with 0.1 m phosphoric acid rinsing solution, followed by fixation in 1% osmium acid for 2 h. The samples were dehydrated with gradient ethanol and then embedded. Ultrathin sections were obtained with an ultrathin sectioning machine (Leica UC7, Leica), and the acquired sections were double stained with 3% uranyl acetate–lead citrate. Subsequently, the morphology of subcellular structures was observed under TEM (HT770, HITACHI).

### mRNA Sequencing and Analysis

MG‐63 cells were seeded in six‐well plates, and when the cells in each well reached 80%, they were treated with PBS and RCMP+US (1.0 W cm^−2^, 1.0 MHz, 5 min, 50% duty cycle), respectively. 24 h of culture was followed by RNA extraction from the samples with TRIzol (Invitrogen) and genomic DNA removal using DNaseI (TaKara). RNA‐seq transcriptome library was established and high‐throughput sequencing was further performed.

### In Vivo Biosafety Evaluation

12 healthy female ICR mice (4–6 weeks) were randomly divided into four groups to evaluate the biosafety of RCMP. Of these, three groups of mice were intravenously injected with 100 µL RCMP (10 mg mL^−1^) on day 7, 14, and 21 prior to execution. The other group of mice was intravenously injected with same volume of saline on day 0. Afterward, blood samples of mice were collected at the end of treatment for further blood routine and blood biochemical examination. Mice in each group were sacrificed and the major organs (heart, liver, spleen, lungs, and kidneys) were collected for analysis of H&E staining.

### In Vivo Distribution Study of Nanoparticles

RCMP and SiO_2_@RCMP labeled with cy5.5 (Ex/Em = 673/707 nm) was intravenously injected into HOS‐MNNG‐bearing xenograft tumor‐bearing mice (*n* = 3, the tumor reached 100 mm^3^). The dose of both RCMP and SiO_2_@RCMP was 5 mg kg^−1^ based on the weight of RCMP. Subsequently, the mice were fully anesthetized by inhalation of a mixture of oxygen with isoflurane (5%) and imaged via PerkinElmer IVIS system at different given times (1, 2, 4, 6, 8, 10, 12, and 24 h) after the injection.

### In Vivo Therapeutic Effects

To obtain the HOS‐MNNG tumor‐bearing mouse model, healthy female Balb/c nude mice (4–6 weeks) were subcutaneously injected into HOS‐MNNG cells (1 × 10^6^ cells dispersed in PBS) in the right hind leg of the mice. When the tumor size reached 50 mm^3^, the mice were randomly divided into five groups (*n* = 5): 1) saline, 2) US irradiation, 3) RCMP, 4) RCP + US, and 5) RCMP+US irradiation (1.0 W cm^−2^, 1.0 MHz, 5 min, 50% duty cycle). The dosage of RCP and RCMP was 10 mg kg^−1^. The various treatments were repeated three times. The body weight and tumor volume (volume [*V*] = length × width^2^/2) of each group of mice were recorded every other day during the 14 days of treatment. Mice were executed at the end of treatment, and tumors and major organs were obtained from each group. The tumors were weighed for each tumor. The collected tumor tissues and major organs were analyzed by H&E staining. In addition, TUNEL and Ki‐67 staining were also used to observe cellular apoptosis after different treatments. The expression levels of GPX4 in tumor tissues of different treatment groups were assessed by GPX4 immunohistochemical staining. Besides, hypoxia and ROS levels in tumor tissues were assessed by HIF‐1*α* and DCFH‐DA immunofluorescence staining, respectively.

### Statistical Analysis

All data were presented as mean ± standard deviation and analyzed using GraphPad Prism 8.0. The significance of the data was evaluated according to the one‐way ANOVA test or two‐way ANOVA. *p < 0.05, **p < 0.01, ***p < 0.001, ****p < 0.0001, ns: not significant.

## Conflict of Interest

The authors declare no conflict of interest.

## Supporting information

Supporting Information

## Data Availability

The data that support the findings of this study are available from the corresponding author upon reasonable request.
