# Supplementary material for: Covalent Organic Framework Nanobowls as Activatable Nanosensitizers for Tumor‐Specific and Ferroptosis‐Augmented Sonodynamic Therapy
Source: Adv Sci (Weinh). 2023 Jan 3;10(6):2206009. doi: 10.1002/advs.202206009 (PMC9951320; doi:10.1002/advs.202206009)
Supplement: Supplementary file 1 — Supporting Information [file ADVS-10-2206009-s001.pdf]

Supporting Information

Covalent Organic Framework Nanobowls as Activatable Nanosensitizers for Tumor-Specific and Ferroptosis-Augmented Sonodynamic Therapy

*Shanshan Zhang, Shujun Xia, Liang Chen *, Yu Chen *, Jianqiao Zhou**

S. Zhang, Dr. S. Xia, Prof. J. Zhou
Department of Ultrasound Ruijin Hospital, Shanghai Jiaotong University School of Medicine, Shanghai 200025, P. R. China
Email: zjq11432@rjh.com.cn

Dr. L. Chen, Prof. Y. Chen
Materdicine Lab, School of Life Sciences, Shanghai University, Shanghai 200444, P. R. China
Email: liangchenbio@shu.edu.cn (Dr. L. Chen); chenyuedu@shu.edu.cn (Prof. Y. Chen).

Supplementary Figures

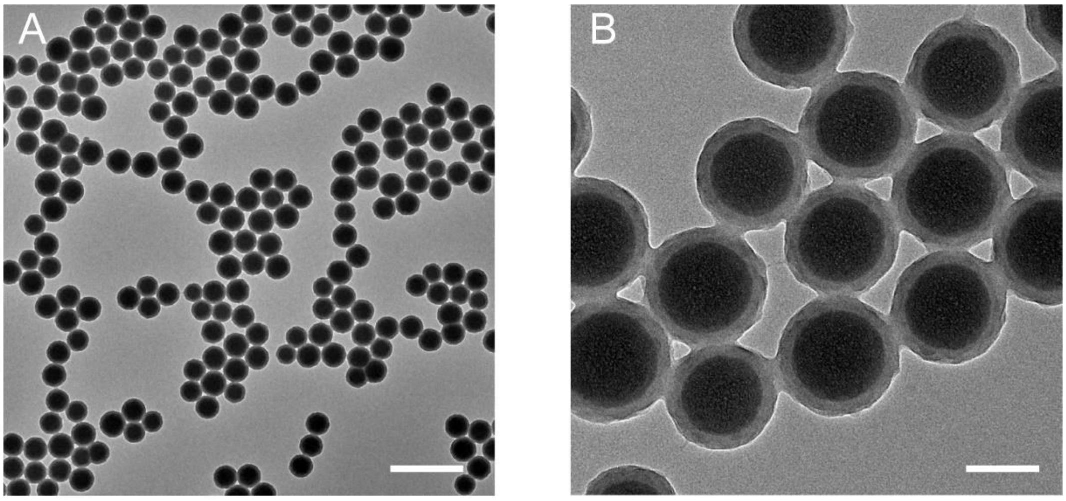

Figure S1. TEM images of SiO_2@COF. Scale bars are 500 nm in A and 100 nm in B. A representative image of three replicates is shown. The SiO_2@COF nanoparticles are very homogeneous and mono-dispersed.

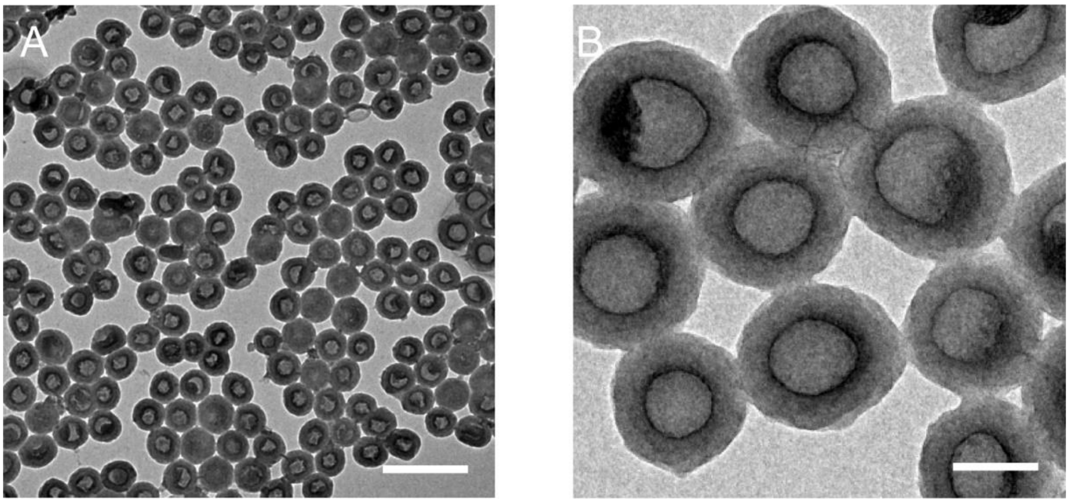

Figure S2. TEM images of the hollow structured COF nanoparticles. Scale bars are 500 nm in A and 100 nm in B. A representative image of three replicates is shown. When the thickness of COF shell layer is too thick, the COF nanoparticles cannot form a bowl-like structure and transform into a hollow structure.

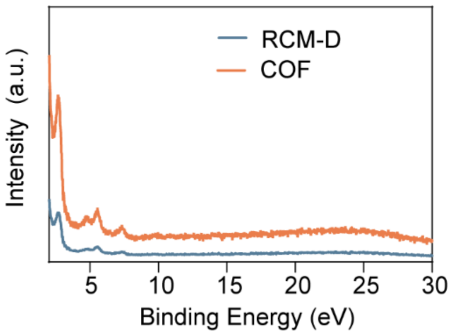

Figure S3. X-ray powder diffraction (XRD) patterns RB@COF-MnO_x obtained by directly oxidized by $KMnO_4$. The intensity of the diffraction peaks of COF decreased remarkably after direct oxidation with $KMnO_4$, which may be due to the strong oxidation of $KMnO_4$ destroying the structure of COF without the protection of dopamine polymerization film.

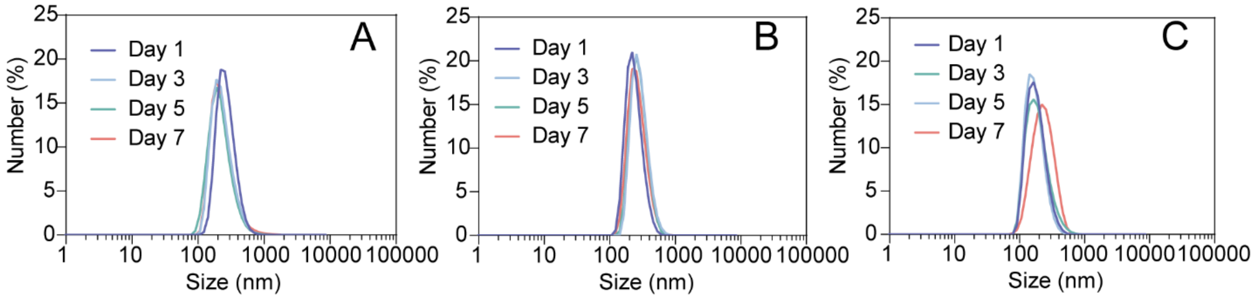

Figure S4. Size distribution of functionalized COF nanobowls in (A) ultrapure water, (B) PBS and (C) cell culture medium after incubation for 1, 3, 5, and 7 days. No obvious agglomeration and precipitation were observed after incubation for different durations under different conditions, indicating that the PEGylated COF-MnO_x feature desirable colloidal stability.

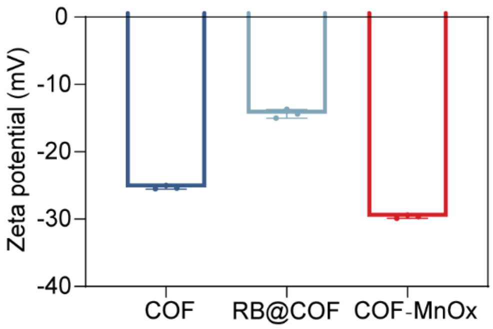

Figure S5. Zeta potential of COF and functionalized COF nanobowls, respectively.

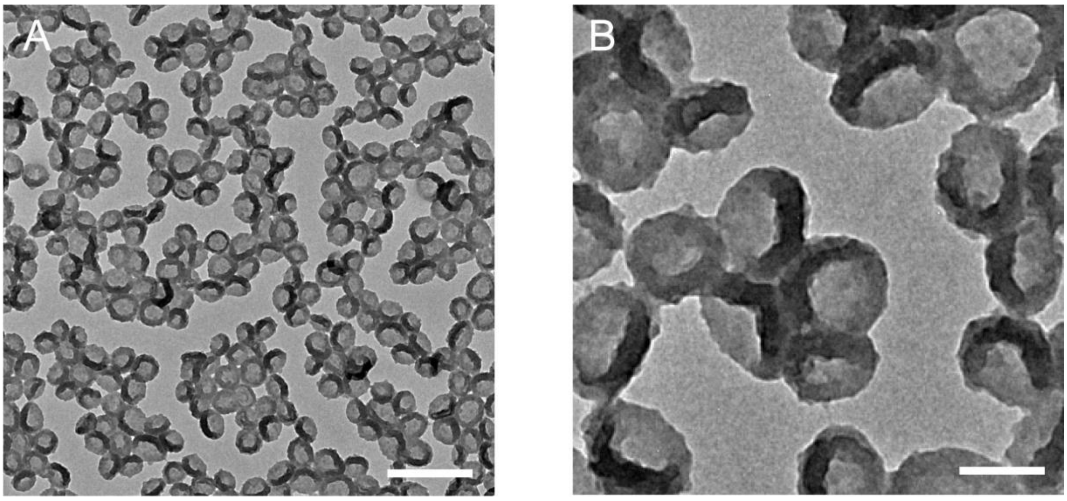

Figure S6. TEM images of RB@COF. Scale bars are 500 nm in A and 100 nm in B. A representative image of three replicates is shown. After loading RB, the nanoparticles still maintain the bowl-shaped morphology.

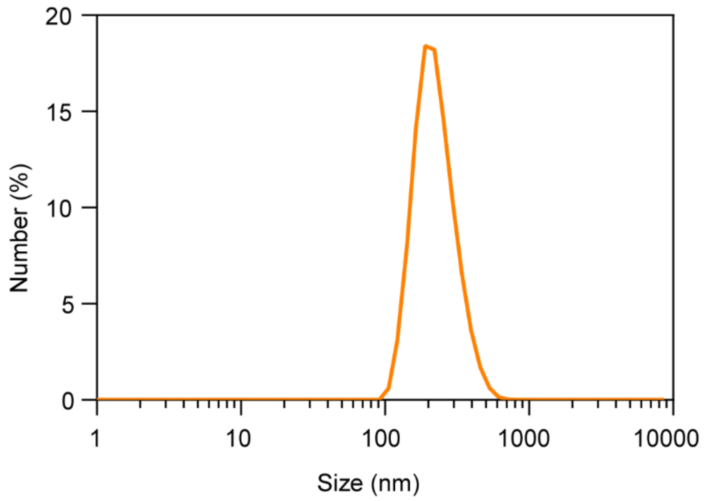

Figure S7. Size distribution of RB@COF. The result shows that there is no significant change in the size of nanobowls after loading RB in the frameworks.

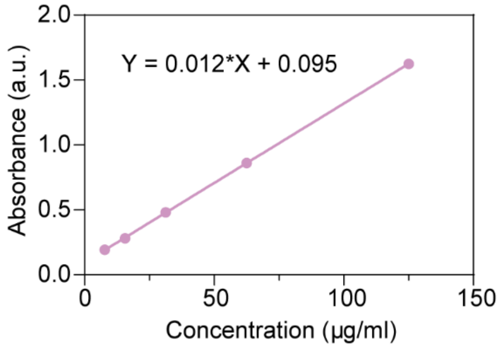

Figure S8. Standard curve for the concentration of RB aqueous solution. The COF is mixed with RB aqueous solution and stirred overnight, and RB can be loaded in COF. After centrifuging the reaction solution, the absorbance of the supernatant was measured. the remaining amount of RB in the supernatant can be obtained from the absorbance and standard curve, and thus the encapsulation rate of RB in COF can be calculated.

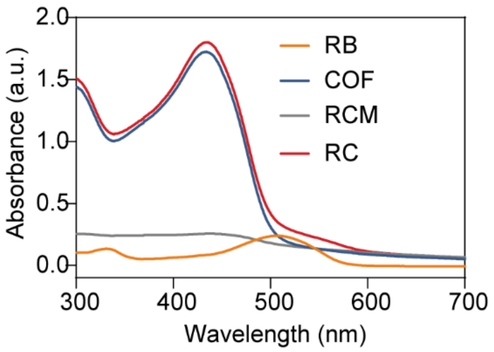

Figure S9. UV absorption spectra of different nanoparticles in ultrapure water. The characteristic absorbance peak of RB at the wavelength of 506 nm is visible on the absorbance curve of RB@COF, which proves the successful loading of RB. However, the characteristic absorption peak of RB disappears when MnO_x is grown in situ, which indicates that the activity of RB can be blocked. (RC: RB@COF; RCM:RB@COF-MnO_x.)

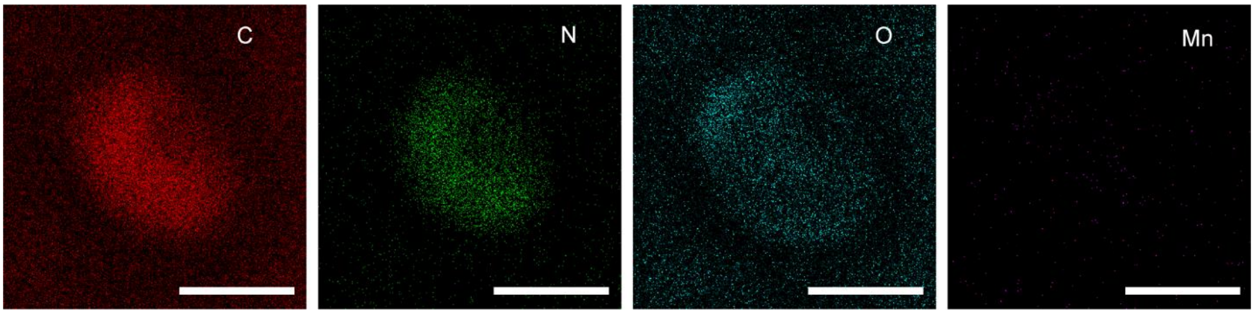

Figure S10. Elemental mapping images of activated nanosensitizer RCMP. All scale bars are 100 nm. After reacting with 10 mM GSH solution, the MnO_x is consumed, resulting in the disappear of Mn in the elemental mapping images.

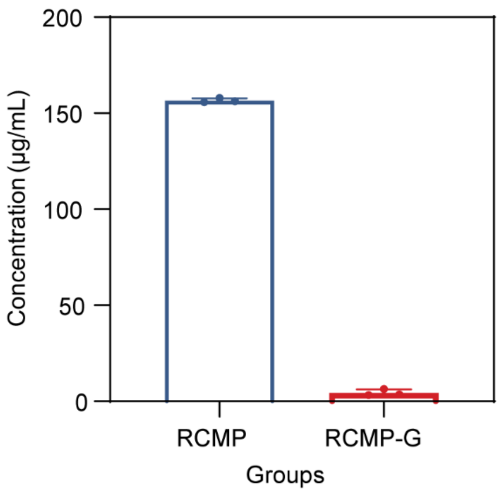

Figure S11. The concentrations of Mn element in RCMP or RCMP-G solution. The RCMP-G represents the GSH-treated RCMP. It is demonstrated that the Mn element is barely detected after reacting with GSH, which further demonstrates the potential of GSH-triggered SDT.

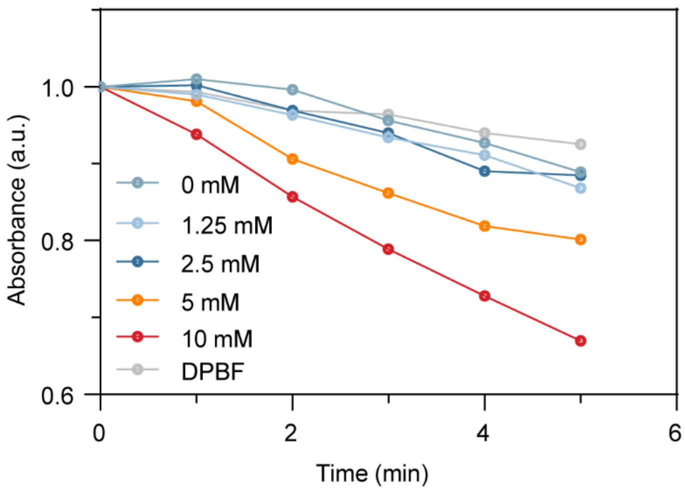

Figure S12. Sonodynamic efficiency of RCMP after incubation with different concentrations of GSH solution. After incubation with GSH solution, the mixture was centrifuged to remove the redundant GSH. Then the DPBF probe was applied to evaluate the sonodynamic efficiency of pretreated-RCMP. It is evident that RCMP can be significantly activated until the concentration of GSH solution reaches 5 mM. As the concentration of GSH is reported to be 10 mM in cancer cells, which is at least 4 times higher than normal cells, RCMP is supposed to be activated in tumor cells only.

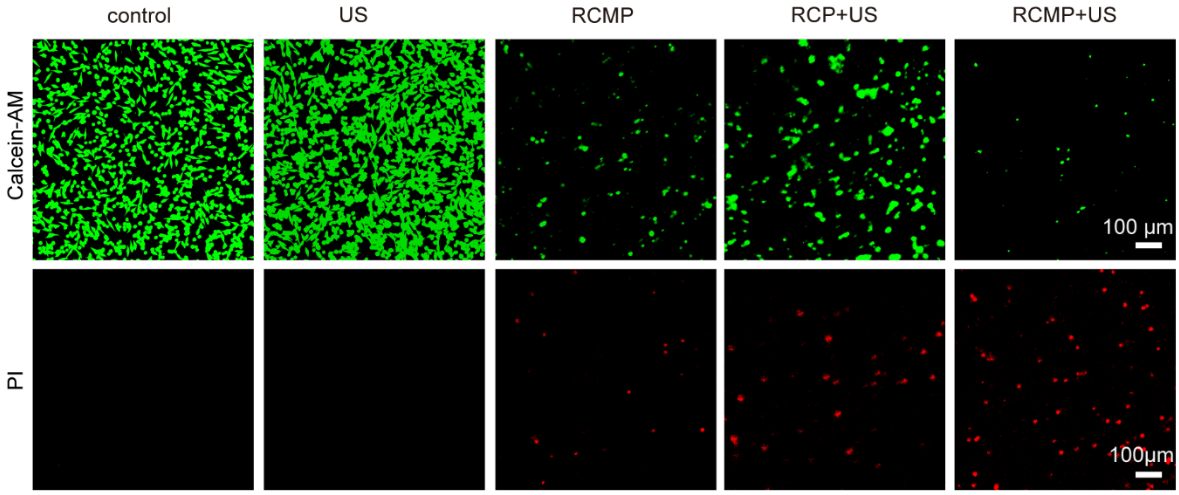

Figure S13. Confocal images of MG-63 cells stained with Calcein-AM/PI for live/dead cells identification after different treatments.

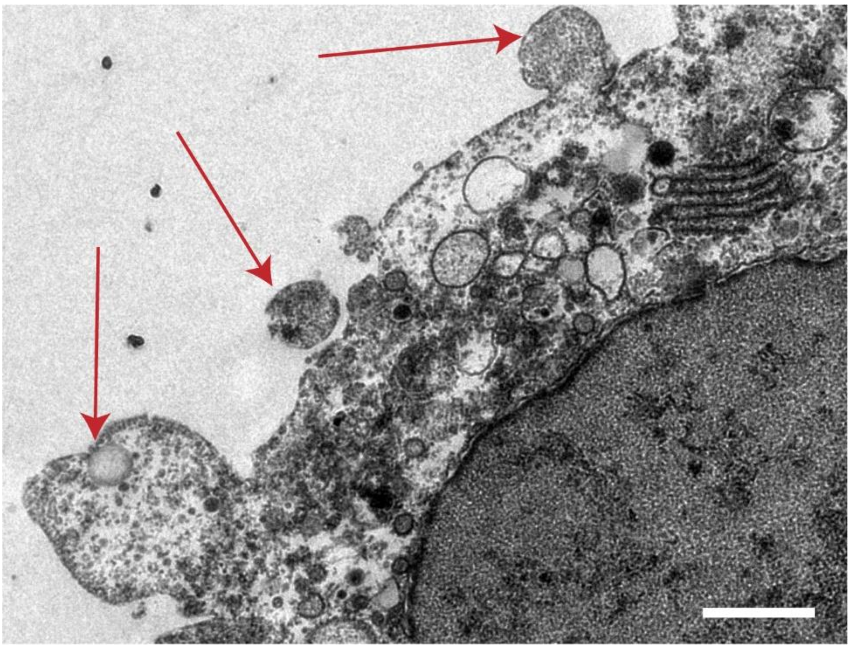

Figure S14. Bio-TEM image of cells treated with RCMP + US irradiation (GSH-activated SDT). The scale bar is 1 μm. It can be observed from the image that cells treated with the RCMP + US exhibits cell membrane discontinuity accompanied by effervescence (red arrows).

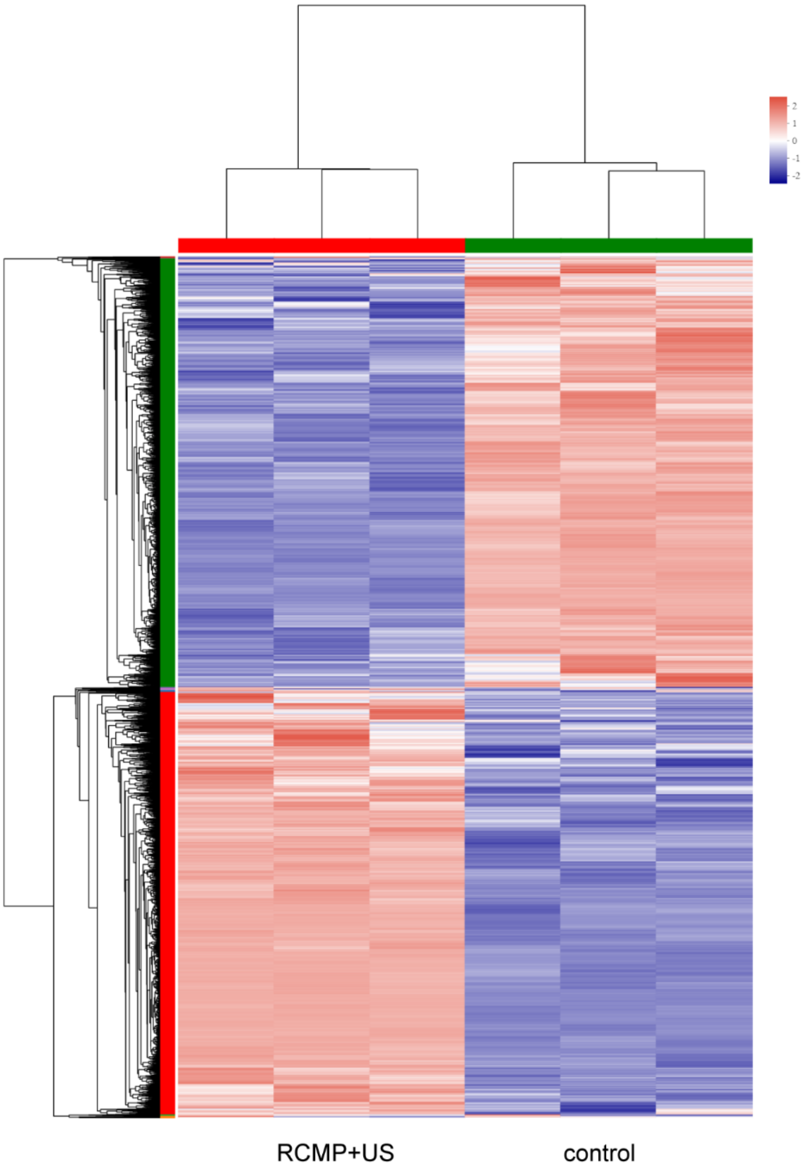

Figure S15. Differentially expressed genes of MG-63 cells screened from RCMP+US and control group (with absolute fold change ≥ 2 and P < 0.05). The cluster analysis reveals significant transcriptomic differences between the control and RCMP+US groups.

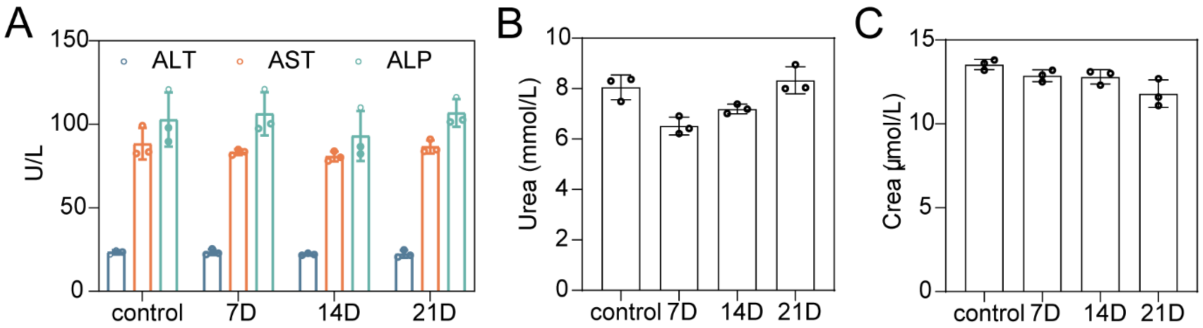

Figure S16. The biochemical parameters of the ICR mice after intravenous injection with saline (control) and the RB@COF-MnO$_x$-PEG nanobowls at the concentration of 10 mg mL^{-1}. At 7, 14 and 21 days after injection, the liver and kidney function indices of the mice were normal.

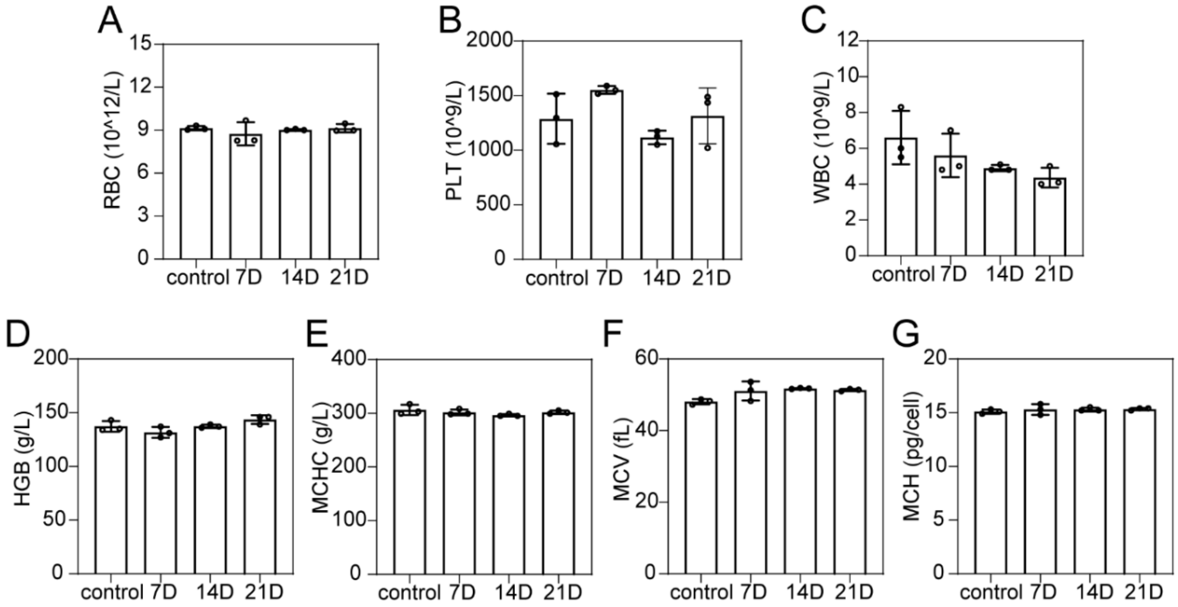

Figure S17. The routine blood indexes of the ICR mice after intravenous injection with saline (control) and the RB@COF-MnO$_x$-PEG nanobowls at the concentration of 10 mg mL^{-1}. At 7, 14 and 21 days after injection, no significant abnormal changes are observed compared with the control.

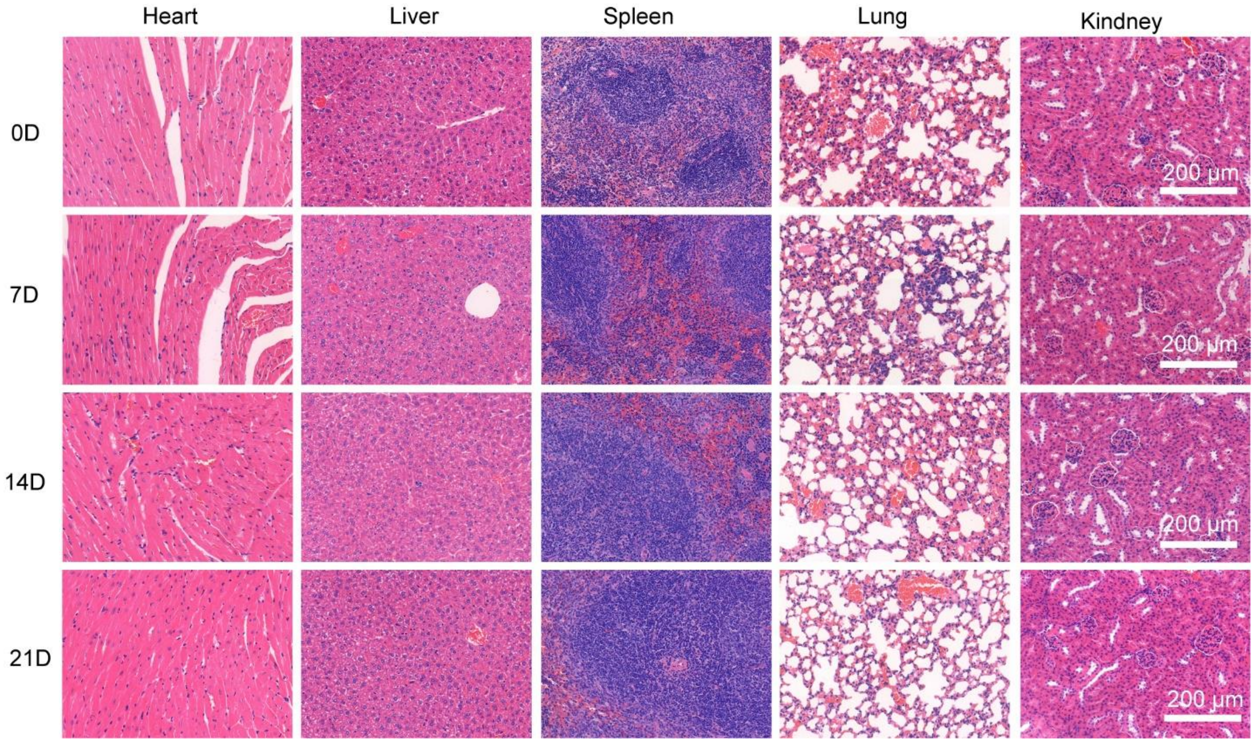

Figure S18. H&E staining images of major organs (heart, liver, spleen, lung and kidney) after intravenous injection of RCMP for different durations. There is no tissue damage observed on histopathological analysis.

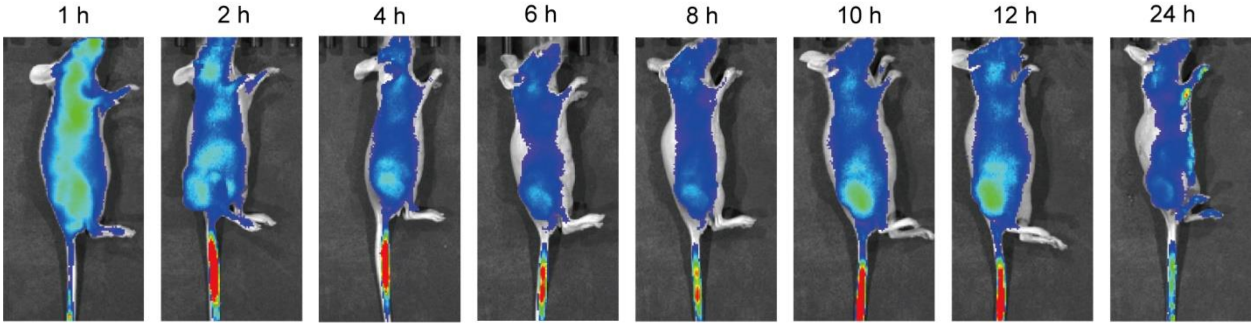

Figure S19. *In vivo* fluorescence images of tumor-bearing mice after the intravenous injection of SiO_2@RMCP-cy5.5 at different time points. The biodistribution of different nanoparticles in mice was investigated using IVIS in vivo optical imaging system. It is found that the fluorescence signal of RMCP at the tumor site was higher than that of SiO_2@RMCP, indicating the bowl-shaped morphology facilitates the enrichment of nanoparticles at the tumor site.

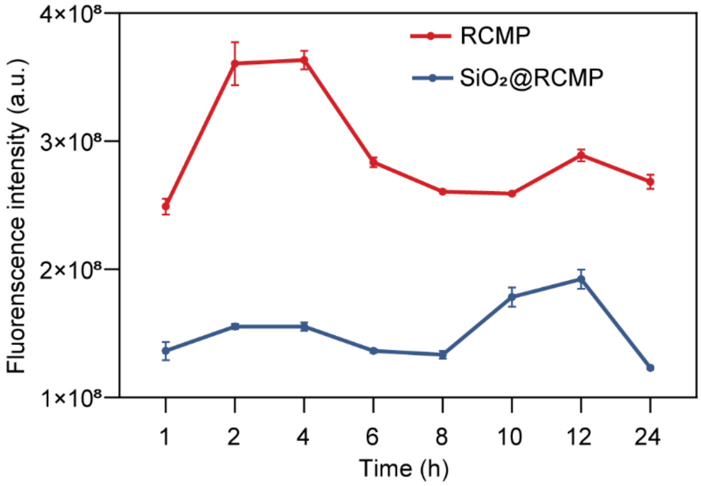

Figure S20. The intensities of the corresponding fluorescent images of RMCP and SiO_2@RMCP groups at different time points (n=3, mean ± s.d.). It is evident that RCMP can accumulate rapidly at the tumor site and maintain a higher fluorescence intensity than its counterpart SiO_2@RMCP in the observation period.

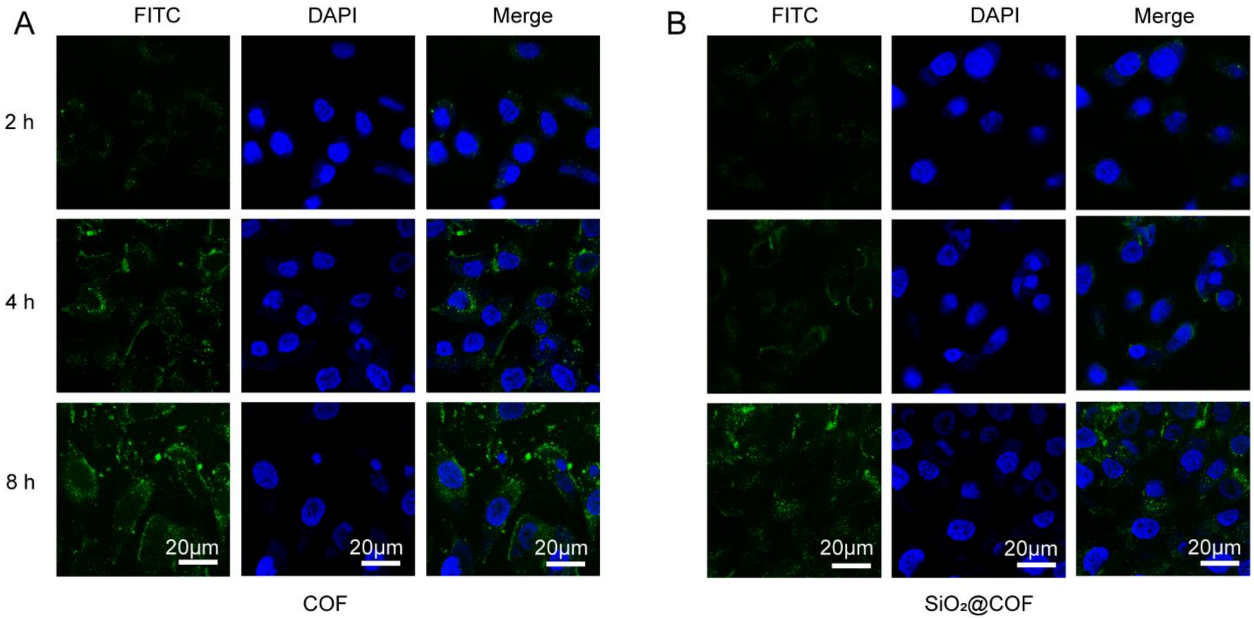

Figure S21. CLSM images of MG-63 cells treated with FITC-labeled COF or SiO_2@COF for 2, 4, or 8 h. Time-dependent confocal images show that MG-63 cells co-incubated with COF exhibit stronger FITC fluorescence compared to SiO_2@COF. Blue and green indicate the nucleus and FITC-labeled nanoparticles, respectively.

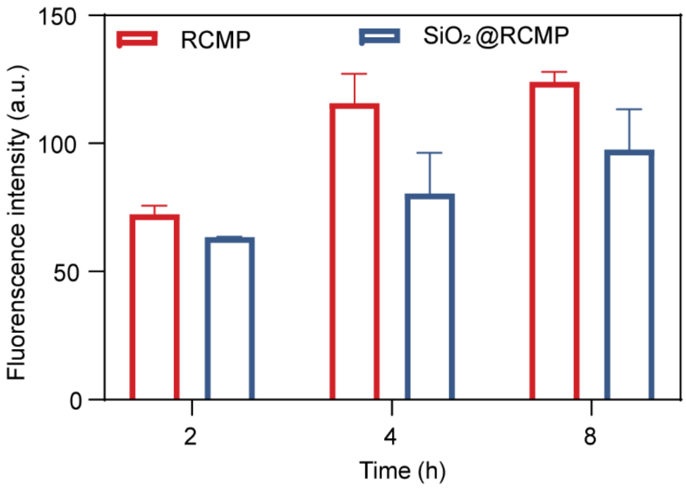

Figure S22. Semi-quantitative results of fluorescence intensity of confocal images in Figure S21. The ImageJ was applied to perform a semi-quantitative analysis of the fluorescence intensity of CLSM images (n=3, mean ± s.d.). The results demonstrate the RCMP has more efficient tumor cell internalization than SiO_2@RCMP, which might be ascribed to the unconventional morphology and reduced stiffness of RCMP.

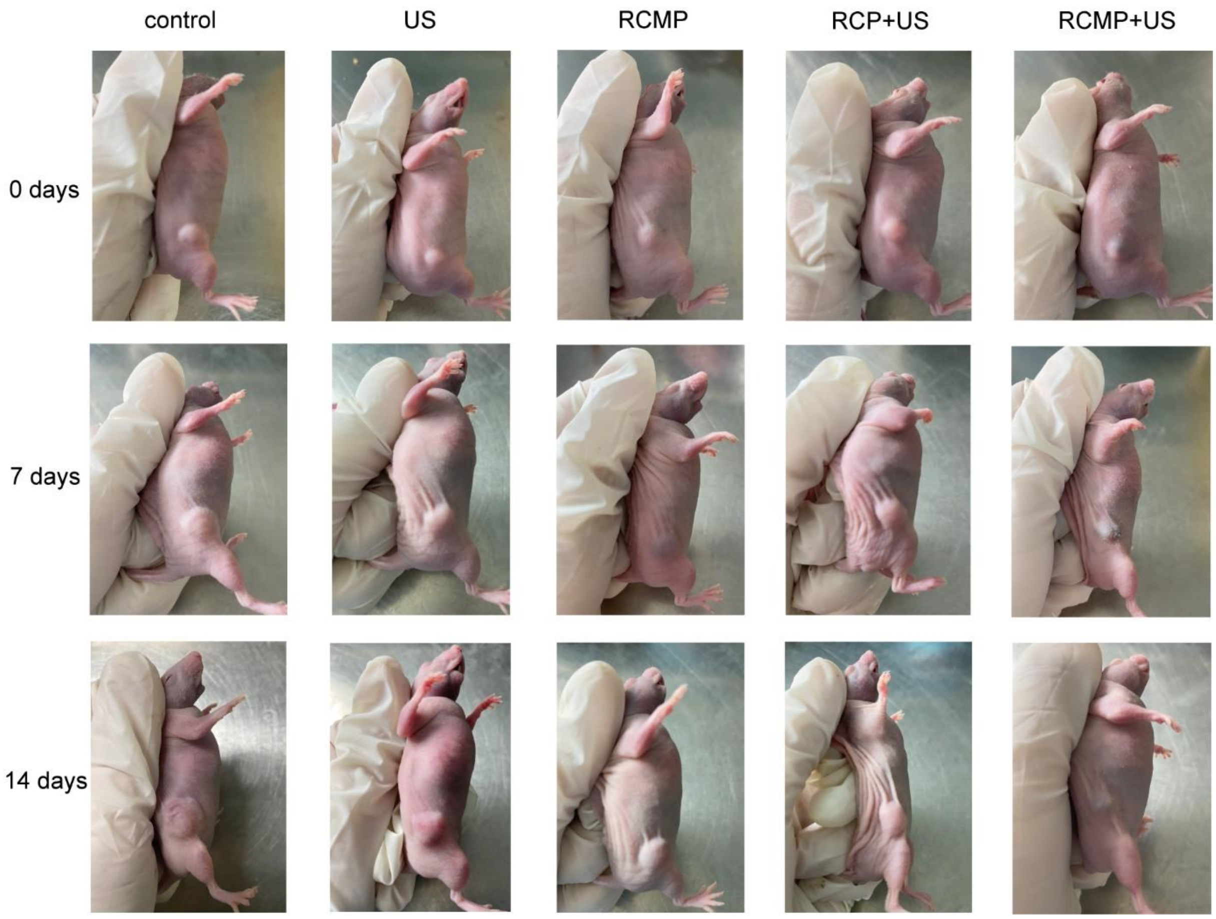

Figure S23. Photographs of osteosarcoma tumor-bearing mice during the 14-day treatment period.

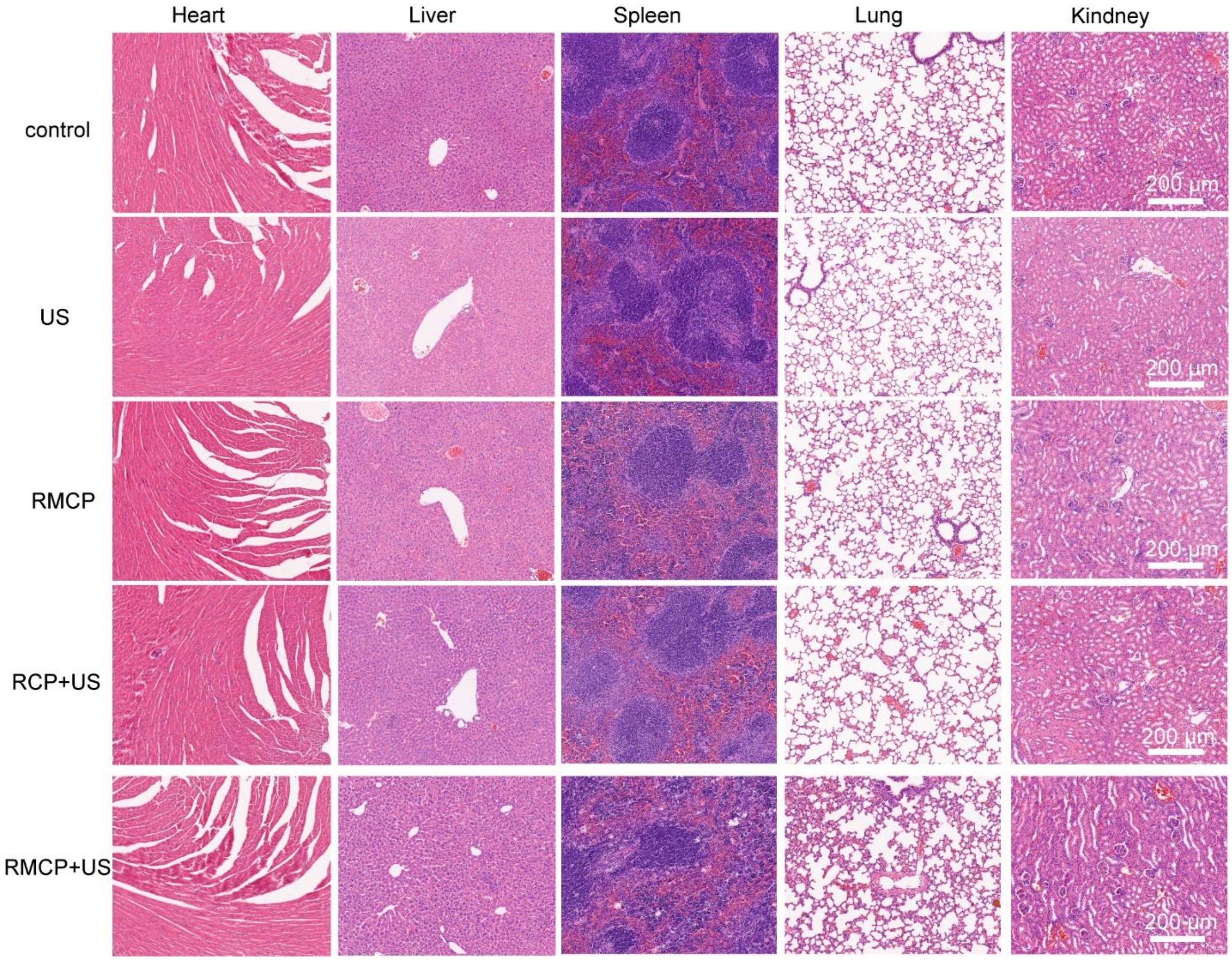

Figure S24. H&E staining of major organs (heart, liver, spleen, lung, and kidney) of differently treated mice for in vivo toxicity evaluation. After 14 days using various treatments, the major organs of mice were collected for hematoxylin-eosin staining (H&E), and no abnormal tissue damage was observed in the major organ tissues in all treatment groups.

Table S1. Apoptosis profiles of MG-63 cells after being treated with various disposes. Compared with the control, the apoptosis ratio of groups treated with RCMP and RCP are evaluated. However, the group treated with RCMP followed by US irradiation induces highest percentage apoptosis cells, indicating the superior efficiency of GSH-activated SDT.

	control	US	RMCP	RCP+US	RMCP+US
Live (%)	96.8	96.8	78.9	87.3	33.9
Apoptosis (%)	1.29	1.27	4.36	7.66	3.09
Late Apoptosis (%)	1.5	1.41	16.2	4.42	61.6
